# CAF-derived exosomal WEE2-AS1 facilitates colorectal cancer progression via promoting degradation of MOB1A to inhibit the Hippo pathway

**DOI:** 10.1038/s41419-022-05240-7

**Published:** 2022-09-19

**Authors:** Peng Yang, Dongsheng Zhang, Tuo Wang, Jiangzhou Ji, Chi Jin, Chaofan Peng, Yuqian Tan, Jiahui Zhou, Lu Wang, Yifei Feng, Yueming Sun

**Affiliations:** 1 Department of General Surgery, The First Affiliated Hospital of Nanjing Medical University, Nanjing, Jiangsu China; 2 The First School of Clinical Medicine, Nanjing Medical University, Nanjing, China; 3 The Colorectal Institute of Nanjing Medical University, Nanjing, China

**Keywords:** Colorectal cancer, Cancer microenvironment

## Abstract

Cancer-associated fibroblasts (CAFs) are the most abundant stromal components in the tumor microenvironment (TME) and closely involved in tumor progression. However, the precise biological functions and molecular mechanisms of CAFs in the TME have yet to be understood. Here, we demonstrate that WEE2-AS1 is highly expressed in the CAF-derived small extracellular vesicles (sEVs). Moreover, WEE2-AS1 is markedly higher in plasma sEVs of CRC patients than in healthy subjects and its high level predicts advanced pathological staging and poor survival. Then, we conducted a series of in vitro and in vivo experiments. Elevated expression of WEE2-AS1 in sEVs increases CRC cell proliferation in vitro. Importantly, aberrant CAF-sEVs^WEE2-AS1^ leads to tumor formation and progression in BALB/c nude mice and promotes AOM/DSS-induced tumorigenesis. Mechanistically, WEE2-AS1 functions as a modular scaffold for the MOB1A and E3 ubiquitin-protein ligase praja2 complexes, leading to MOB1A degradation via the ubiquitin-proteasome pathway. The Hippo pathway is then inhibited and more YAP are transported into the nucleus, where they activate downstream gene transcription. Together, our data reveal that CAF-sEVs^WEE2-AS1^ interacts with MOB1A, promotes degradation of MOB1A, inhibits the Hippo pathway, and facilitates the growth of CRC cells. Hence, exosomal WEE2-AS1 may be a promising therapeutic target and circulating biomarker for CRC diagnosis and prognosis.

## Introduction

Colorectal cancer (CRC) is the third most common malignancy worldwide [1]. Recently, the prognosis of patients has been substantially improved by evolving treatment strategies, including advances in surgical techniques, multiple targeted drugs, but CRC remains a leading cause of death worldwide [2, 3]. The main reason is that the precise mechanisms of tumor initiation and progression remain elusive. Therefore, it is necessary to further elucidate the underlying mechanisms and explore new treatment targets, which are of great value to improve the prognosis of CRC patients.

To date, numerous studies have focused on the characteristics of tumor cells, but the paradigms of cancer-centric therapeutics, which predominantly target the solid tumor cells, can not eradicate or even slow down malignant tumors [4, 5]. Recently, researchers have increasingly realized the importance of the TME in fostering cancer development, which plays the role of “soil” of tumor cell “seed” in CRC and other tumors [6, 7]. TME is mainly composed of fibroblasts, macrophages, and other components [8, 9]. Among all stromal cells, CAFs are the most abundant stromal components. Mechanistically, CAFs build up and remodel the microenvironment, and accelerate tumor progression by secreting growth factors, cytokines, extracellular vesicles, etc [6, 10, 11]. Nevertheless, the communications between CRC cells and CAFs are still unknown.

Small extracellular vesicles (sEVs) are small dual-membrane particles with a diameter of 30–200 nm, released by various cells via fusion with the cell membrane [12]. sEVs play an important role in tumor progression by transporting key molecules, such as cytosolic proteins, lipids, RNAs, etc [13, 14]. On the one hand, CAFs promote cancer progression by transmitting signals through sEVs. For example, CAFs-derived exosomal miR-196a leads to cisplatin resistance [15]. In CRC, CAFs-secreted exosomal miR-92a-3p confers metastasis [16]. On the other hand, cancer cells also could induce the activation and differentiation of fibroblasts. For instance, liver cancer cells-secreted exosomal miR-1247-3p promotes the activation and differentiation of CAFs [17].

In our study, we found that CAF-sEVs promoted the growth of CRC cells. LncRNA WEE2-AS1 was highly expressed in CAF-sEVs compared to NF-sEVs, and elevated expression of WEE2-AS1 in CAF- sEVs could be absorbed by CRC cells and promoted cancer cells growth. Mechanistically, we confirmed that WEE2-AS1 functioned as a modular scaffold for the MOB1A and E3 ubiquitin-protein ligase praja2 complexes, leading to the degradation of MOB1A and inhibition of the Hippo pathway. Furthermore, we also found that WEE2-AS1 was overexpressed in plasma sEVs, and increased WEE2-AS1 expression was correlated with advanced pathological staging and poor outcomes. Overall, WEE2-AS1 enriched in sEVs may be a promising therapeutic target and circulating biomarker for CRC diagnosis and prognosis.

## Results

### CAFs promote the growth of CRC cells via sEVs in vitro

**Fig. 1:**
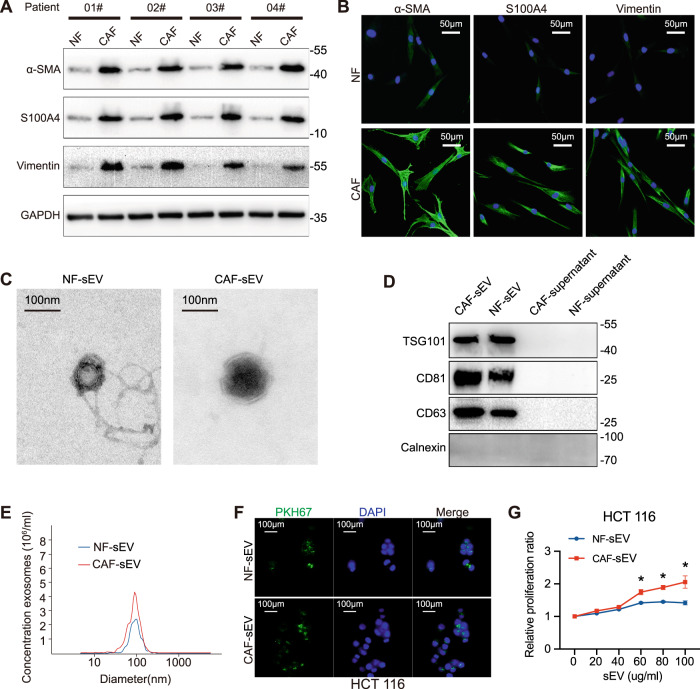
Extraction and identification of fibroblasts and sEVs. **A**, **B** Western blot and immunofluorescence staining for α-SMA, S100A4, and Vimentin expression of NFs and CAFs (scale bar, 50 μm). **C**–**E** Identification of medium sEVs of NFs and CAFs by transmission electron microscopy (scale bar, 100 nm), western blot, and NTA (nanoparticle tracking analysis). **F** sEVs labeling and tracing (scale bar, 100 μm). **G** A CCK8 assay detection of cell viability. Cell proliferation ratio: different *EVs* group*/PBS group*. Data are presented as the mean ± SD. *P < 0.05.

CAFs and NFs were isolated and adherent in culture with a spindle-shaped morphology (Fig. S1A). Specific fibroblast markers (α-SMA, S100A4, and Vimentin) were used to identify the purity and phenotype of the fibroblasts. Results indicated that CAFs had higher expression of specific markers than NFs (Fig. 1A). IF assays further confirmed the cellular morphology and specific markers of CAFs and NFs (Fig. 1B). sEVs in CAFs/NFs -conditioned medium were collected by ultracentrifugation. The membrane-encapsulated structure, diameter, and concentration of sEVs were observed by transmission electron microscopy (TEM) and nanoparticle tracking analysis (NTA) (Fig. 1C, E). sEVs displayed closed round vesicles with diameters of 100 nm. In addition, we characterized sEVs by the positive markers TSG101, CD81, and CD63, while Calnexin was used as a negative marker (Fig. 1D). Then, CRC cells were incubated with PKH67 (green)-labeled sEVs, and subsequently, the recipient cells were observed by a confocal microscope. Results revealed that green spots (PKH67-labeled sEVs) localized inside of recipient CRC cells, suggesting labeled sEVs released by CAFs/NFs were transferred to CRC cells (Figs. 1F, S1B). We next evaluated whether CAF/NF-sEVs participated in the progression of CRC. CRC cells were co-cultured with CAF/NF-sEVs on different concentrations (0, 20, 40, 60, 80, or 100 μg/ml) for 24 h. As shown in Figs. 1G and S1C, CAF-sEVs significantly promoted CRC cells proliferation when the concentration of sEVs was higher than 60 and 40 μg/ml. Thus, CRC cells were then incubated with 60 μg/ml sEVs secreted by CAFs/NFs for 24 h in the next study.

**Fig. 2:**
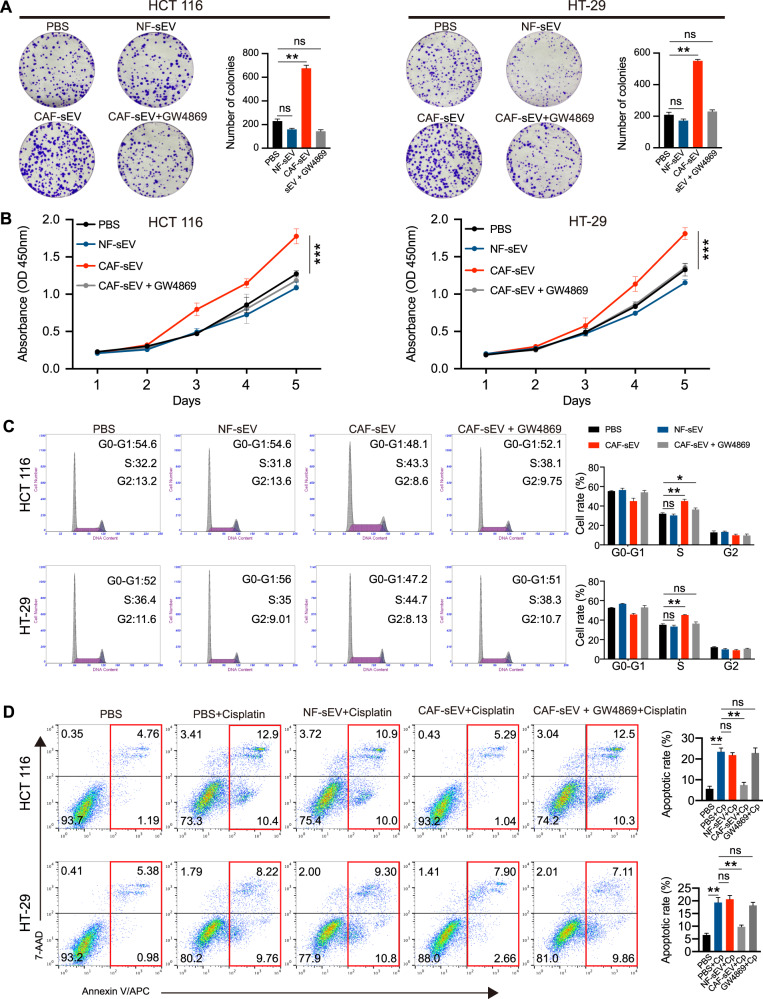
CAFs-derived sEVs promote the growth of CRC cells in vitro. **A** Assessment of the viability of HCT 116 and HT-29 cells co-cultured with sEVs by colony formation assays. **B** CCK8 assays are used to determine the proliferation of CRC cells co-cultured with sEVs. **C** Flow cytometry shows significant increases or decreases in the proportion of cells in S or G1-phase, respectively when HCT 116 and HT-29 cells are co-cultures with CAF-sEVs. **D** Cells were treated with cisplatin (5ug/ml for 24 h). Flow cytometry is used to detect the apoptotic rates (LR + UR) of cells. LR, early apoptotic cells; UR, terminal apoptotic cells. Data are shown as mean ± SD of three independent experiments, *P < 0.05, **P < 0.01, ***P < 0.001, ns. not significant.

In colony formation and CCK-8 assay, after co-cultured with NF-CM, CAF-CM, CAF-sEV, and CAF-CM-depleted of sEV, we confirmed that CAF-sEV participated in the progression of CRC (Fig. S2A, B). Furthermore, the colony formation of CRC cells was significantly increased in the CAF-sEVs treated group compared to the control groups (Fig. 2A). Furthermore, CCK-8 assays similarly indicated that CRC cells of the CAF-sEVs group have higher proliferation than the PBS and NF-sEVs groups (Fig. 2B). Given that cell cycle and apoptosis are two factors in cell proliferation, we examined the changes in cell cycle and apoptosis rate in each group by flow cytometric assays. As shown in Fig. 2C, an increased percentage of S phase populations were consistently observed in the CAF-sEVs group. Additionally, CRC cells incubated with CAF-sEVs had lower apoptotic rates than those in the PBS and NF-sEVs groups (Fig. 2D). Moreover, with the treatment of GW4869 [18, 19], an inhibitor of sEVs internalization, CAF-sEVs failed to promote the ability of colony formation of CRC cells (Fig. 2A). In addition, the promoted effect on CRC proliferation was also disappeared (Fig. 2B). Consistently, the increased S phase and decreased apoptotic rates in CRC cells treated with CAF-sEVs were abolished by GW4869 (Fig. 2C, D). These findings demonstrated that CAF-sEVs increased CRC cells proliferation and inhibited apoptosis stimulated by cisplatin in vitro.

### LncRNA WEE2-AS1 is highly expressed in CAF-sEVs and increases CRC cell growth in vitro

**Fig. 3:**
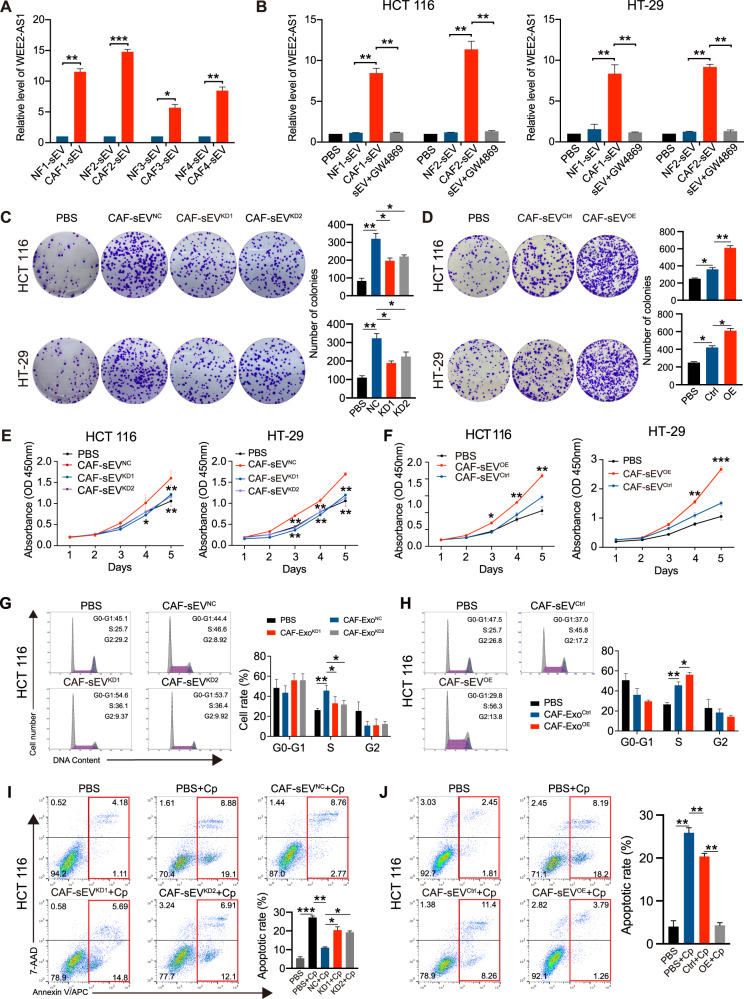
Effect of sEVs WEE2-AS1 on CRC cellular phenotype. **A** Level of WEE2-AS1 in four paired NF-sEVs and CAF-sEVs, as determined by qRT-PCR. **B** Level of WEE2-AS1 in HCT 116 and HT-29 cells are co-cultures with PBS, NF-sEVs, CAF-sEVs, and CAF-sEVs +GW4869, as determined by qRT-PCR. **C**, **D** Assessment of the viability of HCT 116 and HT-29 cells co-cultured with sEVs by colony formation assays. **E**, **F** CCK8 assays are used to determine the proliferation of CRC cells co-cultured with sEVs. **G**, **H** Flow cytometry showing significant decreases or increases in the proportion of cells in S or G1-phase, respectively, when HCT 116 cells co-cultured with CAF-sEVs^KD^ or CAF-sEVs^OE^. **I**, **J** Cells were treated with cisplatin (5ug/ml for 24 h). Flow cytometry is used to detect the apoptotic rates (LR + UR) of cells. LR, early apoptotic cells; UR, terminal apoptotic cells. Data are shown as mean ± SD of three independent experiments, *P < 0.05, **P < 0.01, ***P < 0.001.

sEVs are dual-membrane-encapsulated particles that are abundant in RNA molecules, including miRNAs, circRNAs, and lncRNAs. Therefore, we hypothesized that sEVs RNA molecules possibly were an essential component of cell-cell communication. After analyzing the previous research on sEVs from paired NFs and CAFs, we detected the differential expression lncRNAs and miRNAs in the sEVs derived from CAFs and NFs of four patients [20]. As shown in Figs. 3A and S3A, WEE2-AS1 changed markedly in CAF-sEVs corresponding to NF-sEVs among all RNA molecules. Expectedly, after co-cultured with CAF-sEVs, the levels of WEE2-AS1 expression in CRC cells were significantly increased. The increased effect was suppressed by GW4869 (Fig. 3B). The results showed that WEE2-AS1 was enriched in CAF-sEVs and could be transferred from CAFs to CRC cells. We next established WEE2-AS1-overexpress/knockdown CAFs to assess the functional effect of CAF-sEVs^WEE2-AS1^ on CRC cells (Fig. S4A, D). We further detected the levels of WEE2-AS1 expression in CAF-sEVs. The results indicated that knockdown (KD) or overexpression (OE) of WEE2-AS1 in CAFs decreased or increased the levels of WEE2-AS1 in CAF-sEVs compared with the control groups (Fig. S4B, E). Additionally, after being co-incubated with CAF-sEVs^KD^ and CAF-sEVs^OE^, levels of WEE2-AS1 in CRC cells were also decreased or increased compared with control groups, respectively (Fig. S4C, F). As shown in (Fig. 3C, D), the ability of colony formation of CRC cells in CAF-sEV group was increased compared with that of the PBS group, but the magnitude of the increase was significantly smaller in the CAF-sEV^KD^ group than in the CAF-sEV^NC^ group and larger in the CAF-sEVs^OE^ group than that of the CAF-sEV^Ctrl^ group. CCK-8 assays had similar results (Fig. 3E, F). Flow cytometric assays revealed that CAF-sEV increased S phase populations in CRC cells compared with the PBS group (Figs. 3G, H, S5A, B). The CAF-sEV^KD^ group showed a smaller increase in the proportion of S-phase compared with the CAF-sEV^NC^ group (Figs. 3G, S5A). The increase was significantly greater in the CAF-sEVs^OE^ group than in the CAF-sEV^Ctrl^ group (Figs. 3H, S5B). In apoptosis assays, cisplatin (5ug/ml for 24 h) was used to stimulate apoptotic production. The apoptotic rates in the all sEV groups were lower that the PBS^cisplatin^ group(Figs. 3I, J, S5C, D). Corresponding to the above results, the CAF-sEV^KD^ group showed a smaller decreased apoptotic rates compared with the CAF-sEV^NC^ group (Figs. 3I, S5C), meanwhile the CAF-sEV^OE^ group showed a larger decreased apoptotic rates compared with the CAF-sEV^Ctrl^ group (Figs. 3J, S5D). The increase was significantly greater in the CAF-sEVs^OE^ group than in the CAF-sEV^Ctrl^ group (Figs. 3H, S5B). Overall, these findings indicate that WEE2-AS1 in CAF-sEVs mediates CRC cell proliferation and inhibited apoptosis stimulated by cisplatin in vitro.

### WEE2-AS1 is predominantly localized in the cytoplasm and directly interacts with MOB1A

**Fig. 4:**
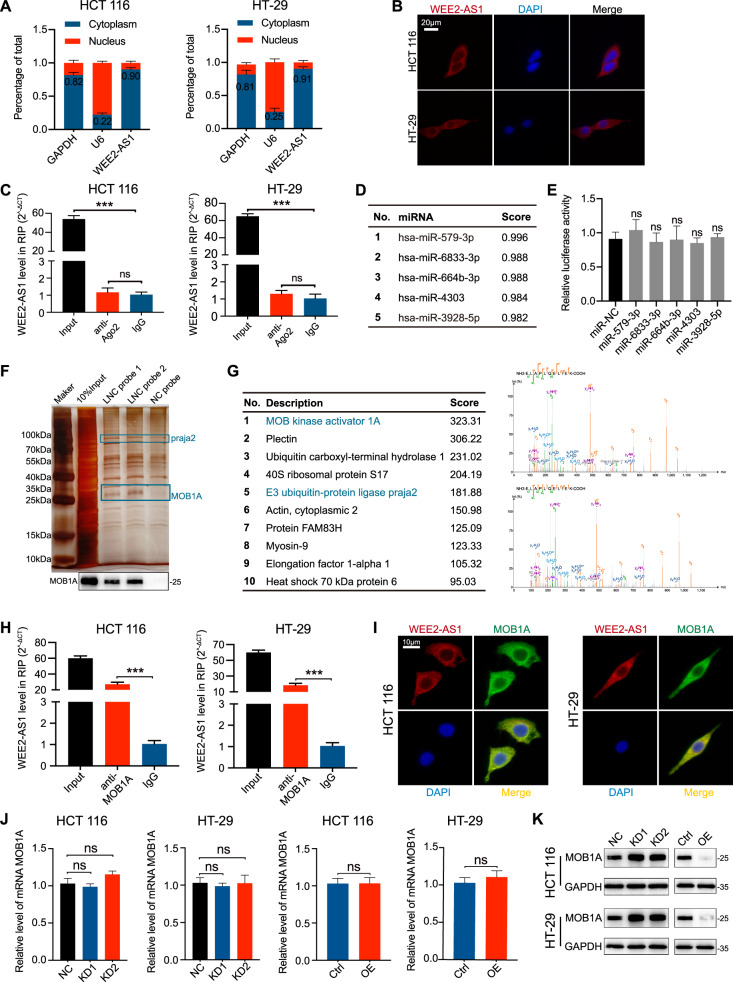
WEE2-AS1 is predominantly localized in the cytoplasm and directly interacts with MOB1A. **A**, **B** Subcellular fractionation assays and RNA-FISH confirm that WEE2-AS1 is predominantly located in the cytoplasm (scale bar: 20μm). **C** Level of WEE2-AS1 detected by qRT-PCR after RIP for Ago2 in HCT 116 and HT-29 cells. **D** The top five miRNAs are predicted from LncBase Experimental v.2. **E** The luciferase reporter plasmid (RLuc-WEE2-AS1) was cotransfected into HEK-293T cells with the 5 various miRNA-coding plasmids. **F** Silver staining of WEE2-AS1 pulldown. Blue boxes show different bands between the WEE2-AS1 and NC probe. **G** List of the top 10 differentially expressed proteins identified by mass spectrometry. The amino acid sequence of a doubly charged peptide was identified as NH2-ELAPLQELIEK-COOH, and a Mascot search showing the peptide matched with MOB1A. **H** Level of WEE2-AS1 detected by qRT-PCR after RIP for MOB1A in HCT 116 and HT-29 cells. **I** Dual RNA-FISH and immunofluorescence assays: WEE2-AS1(red), MOB1A(green) (scale bar: 10 μm). **J**, **K**. qRT-PCR and western blot for MOB1A expression. Data are shown as mean ± SD of three independent experiments, ***P < 0.001, ns. not significant.

To investigate the underlying mechanism by which WEE2-AS1 promotes the growth of CRC cells, we first confirmed that WEE2-AS1 was localized in the cytoplasm (Fig. 4A, B). The most well-known mechanism of cytoplasmic lncRNAs is competitive endogenous RNAs (ceRNAs), which sponge various miRNAs to dampen their regulatory effects on target mRNAs [21, 22]. Further, argonaute 2 (Ago2) is a component of the RNA-induced silencing complex (RISC) involved in miRNA-mediated repression of mRNAs [23, 24]. However, RIP assays revealed that WEE2-AS1 could not be enriched in the anti-Ago2 group (Fig. 4C). This finding probably suggested that WEE2-AS1 can not function as a ceRNA of miRNAs. Online bioinformatics database (LncBase Experimental v.2) was also applied to evaluate miRNAs that have potential binding sites for WEE2-AS1. As shown in Fig. 4D, the top five miRNAs could have potential binding sites for WEE2-AS1. Then, dual-luciferase reporter assays were used to confirm which miRNA could bind to WEE2-AS1. HEK-293T cells were cotransfected with a luciferase plasmid harboring the sequence of WEE2-AS1 together with plasmids encoding the miRNAs or a control sequence. The results showed that WEE2-AS1-driven luciferase activity had no difference between miRNA and NC groups (Fig. 4E). In this case, we confirmed that no miRNAs could be the candidate targets for WEE2-AS1, and WEE2-AS1 can not function as a ceRNA of miRNAs.

Another important mechanism of lncRNAs is interaction with RNA-binding proteins (RBPs) [22]. RNA pulldown assay was conducted by using biotinylated WEE2-AS1 probes and the silver staining showed that several specific bands of proteins were observed in the WEE2-AS1 probes group compared to the control (Fig. 4F). A mass spectrometry assay revealed differential proteins between the WEE2-AS1 probes and NC probe group (Table S4). Figure 4G displayed the top ten differential expressed proteins and the top of the list was MOB kinase activator 1A (MOB1A). WB also showed that MOB1A could be detected in the input group and WEE2-AS1 probes pulldown products, but not in the NC probe pulldown product (Fig. 4F). Then, RIP assay confirmed that WEE2-AS1 could be enriched by anti-MOB1A (Fig. 4H). Furthermore, IF staining assay showed that WEE2-AS1 and MOB1A mainly co-localized in the cytoplasm (Fig. 4I). However, there was no regulatory relationship at the RNA level (Fig. 4J). Unexpectedly, overexpression of WEE2-AS1 inhibited the protein level of MOB1A compared with the control group, while inhibition of WEE2-AS1 promoted MOB1A protein expression (Fig. 4K). Taken together, these findings suggested that WEE2-AS1 interacted with MOB1A and regulated its protein expression level.

### WEE2-AS1 mediates the proteasomal degradation of MOB1A and inhibits the Hippo pathway

**Fig. 5:**
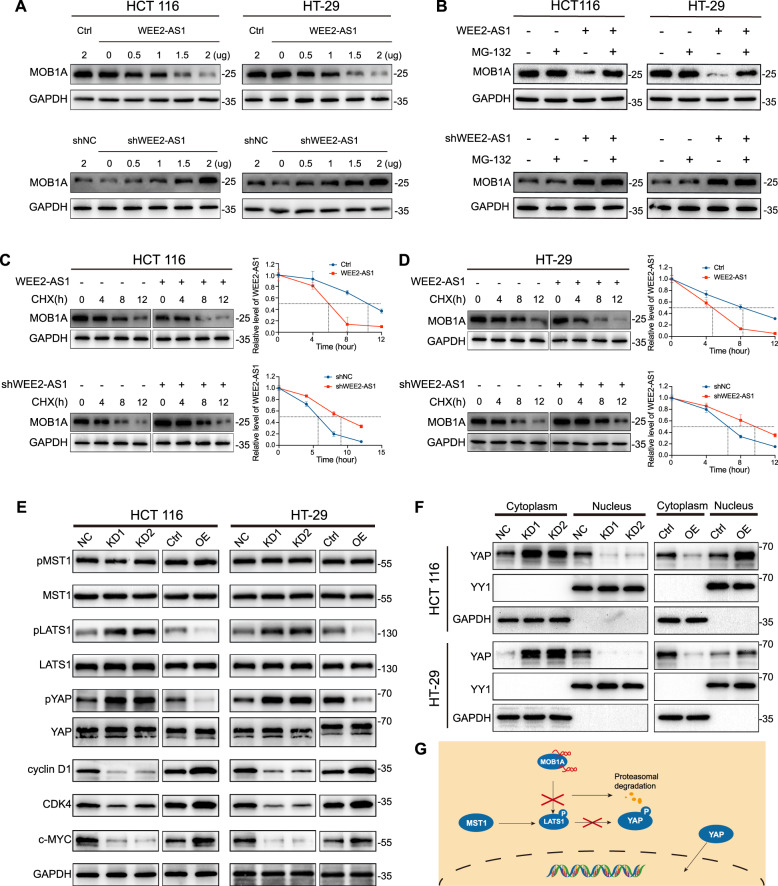
WEE2-AS1 mediates the proteasomal degradation of MOB1A and inhibits the Hippo pathway. **A** HCT 116 and HT-29 cells are transfected with increasing amounts of MOB1A overexpression or knockdown plasmids, and endogenous protein levels of MOB1A were determined by WB. **B** Immunoblotting showing reversion of MOB1A protein levels by proteasome inhibitor MG132 in the WEE2-AS1 knockdown or overexpression HCT 116 and HT-29 cells. **C**, **D** Immunoblotting showing the effect of WEE2-AS1 expression changes on MOB1A protein stability in the HCT 116 and HT-29 cells treated with 100 μg/ml cycloheximide (CHX) for indicated times. **E** Representative western blot analyses of Hippo signaling pathway factors pMST1, MST1, pLATS1, LATS1, pYAP, YAP, c-MYC, cyclin D1, and CDK4 using specific antibodies through gain or loss of function of WEE2-AS1 in HCT 116 and HT-29 cells. GAPDH acts as an internal reference. **F** Level of YAP detected by WB in cytoplasmic and nuclear extraction. YY1 was used as a nuclear internal reference, and GAPDH was used as a plasma reference. **G** Working model for WEE2-AS1 inhibiting the Hippo pathway. Data are shown as mean ± SD of three independent experiments.

To explore the molecular mechanisms by which WEE2-AS1 regulated MOB1A protein levels, we transfected different doses of plasmids into CRC cells and examined the changes in MOB1A protein levels. The results indicated that overexpression of WEE2-AS1 significantly reduced the protein level of endogenous MOB1A in a dose-dependent manner, while knockdown of WEE2-AS1 had the opposite result (Fig. 5A). Furthermore, in WEE2-AS1 overexpressing cells, the reduction of MOB1A protein was specifically reversed by treatment with the proteasome inhibitor MG132, whereas knockdown of WEE2-AS1 had the opposite result (Fig. 5B). The above results suggested that the effect of WEE2-AS1 on MOB1A degradation is mediated by the proteasomal protein-degradation pathway. Next, we performed a cycloheximide chase assay to analyze the effect of WEE2-AS1 on the stability of MOB1A protein. As shown in Fig. 5C, D, MOB1A was rapidly degraded in WEE2-AS1 overexpression cells, while WEE2-AS1 knockdown prolonged the half-life of MOB1A protein degradation, indicating that WEE2-AS1 strictly controlled MOB1A protein stability. Since MOB1A is a key effector of the Hippo pathway, then, we examined the activation of the Hippo pathway in WEE2-AS1 knockdown or overexpression CRC cells. The results indicated that knockdown of WEE2-AS1 promoted phosphorylation of LATS1, and YAP, whereas WEE2-AS1 overexpression had the opposite effect. However, there was no change on p-MST1, total MST1, and total LATS1, or total YAP, which indicated that WEE2-AS1 inhibited the Hippo signaling pathway (Fig. 5E). When Hippo signaling is inactivated, YAP/TAZ enter the nucleus, bind to the transcription factor TEADs, and induce gene transcription. Thus, we isolated nuclear and cytoplasmic proteins from WEE2-AS1 knockdown or overexpressed CRC cells and examined the distribution of YAP. As shown in Fig. 5F, overexpression of WEE2-AS1 promoted the accumulation of YAP in the nucleus, whereas knockdown of WEE2-AS1 had the opposite effects. We also observed that the expression of anti-apoptotic and pro-proliferation genes (c-MYC, cyclin D1, and CDK4) were upregulated in WEE2-AS1 overexpression cells, whereas shWEE2-AS1 cells had the opposite effect (Fig. 5E). These data showed that WEE2-AS1 accelerated MOB1A proteasomal degradation and further inhibited the Hippo signaling pathway which promoted the translocation of YAP into the nucleus.

### WEE2-AS1 regulates MOB1A proteasomal degradation by enhancing its binding to E3 ligases praja2

**Fig. 6:**
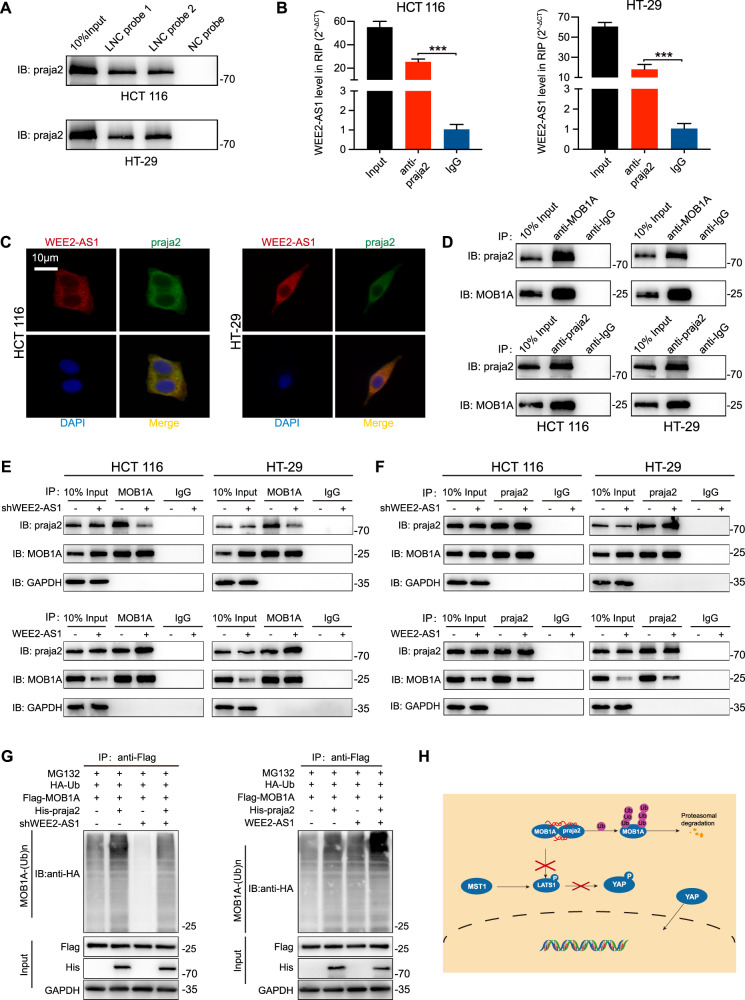
WEE2-AS1 regulates MOB1A proteasomal degradation by enhancing its binding to E3 ligases praja2. **A** Level of praja2 detected by WB in RNA pulldown products. **B** Level of WEE2-AS1 detected by qRT-PCR after RIP for praja2 in HCT 116 and HT-29 cells. **C** Dual RNA-FISH and immunofluorescence assays: WEE2-AS1 (red), praja2 (green) (scale bar: 10 μm). **D** Coimmunoprecipitation and Western blot assay indicate the interaction between MOB1A and praja2 in HCT 116 and HT-29 cells. **E** praja2 immunoprecipitated using an anti-MOB1A antibody in WEE2-AS1 knockdown or overexpression cells. **F** MOB1A immunoprecipitated using an anti-praja2 antibody in WEE2-AS1 knockdown or overexpression cells. **G** HCT 116 and HT-29 cells with WEE2-AS1 knockdown or overexpression were transfected with the indicated constructs and the cells then were treated with MG132 (20 μM) for 8 h before collection. The whole-cell lysate was subjected to immunoprecipitation with anti-Flag antibody and western blot with anti-HA antibody to detect ubiquitylated MOB1A. **H** Working model for WEE2-AS1 mediating MOB1A degradation through proteasomal degradation. Data are shown as mean ± SD of three independent experiments. ***P < 0.001.

There are many key regulators in the progression of the proteasomal protein degradation pathway, such as ubiquitin, E1 ubiquitin-activating enzyme, E2 ubiquitin-conjugating enzyme, E3 ubiquitin-protein ligase, etc [25, 26]. We then re-analyzed the mass spectrometry results and found that E3 ubiquitin-protein ligase praja2 was also enriched in the WEE2-AS1 pulldown products (Fig. 4F, G). Recent studies on praja2 have shown that it mediated MOB1A proteolysis and attenuated Hippo signaling [27]. Additionally, Kindlin-2 constitutes a bridge between praja2 and MOB1 and enhances their interaction to promote the degradation of MOB1 [28]. However, the interaction of praja2 and MOB1A in colorectal cancer was almost unknown. Then, RNA pulldown and RIP assays confirmed that WEE2-AS1 could directly bind to praja2 (Fig. 6A, B). Furthermore, IF staining showed that WEE2-AS1 and praja2 mainly co-localized in the cytoplasm (Fig. 6C). Thus, we hypothesized that WEE2-AS1 may function as a modular scaffold for the MOB1A and praja2 complexes. Next, the associations of proteins MOB1A with praja2 in CRC cells were confirmed by coimmunoprecipitation (Fig. 6D). In addition, We found that WEE2-AS1 depletion drastically decreased MOB1A-praja2 complex formation, whereas WEE2-AS1 overexpression enhanced it. As shown in Fig. 6E, F, anti-MOB1A co-IP assays indicated that more praja2 proteins were enriched in the WEE2-AS1 overexpression group while WEE2-AS1 knockdown weaken the binding. Similar results could also be detected in anti-praja2 co-IP assays. To further find out whether the proteasomal pathway is the underlying mechanism for the degradation of MOB1A, Flag-tagged MOB1A, His-tagged praja2, HA-tagged ubiquitin (Ub), WEE2-AS1 overexpression/knockdown plasmids were cotransfected into HEK293T cells. Then, co-IP assays with anti-Flag antibodies were carried out and immunoblotting for HA-Ub-conjugated proteins. The results revealed that MOB1A was more or less ubiquitylated in WEE2-AS1 overexpression or knockdown group than the control group, respectively (Fig. 6G). These findings indicated that WEE2-AS1 regulates MOB1A proteasomal degradation by enhancing its binding to E3 ligases praja2 (Fig. 6H).

### CAF-sEVs^WEE2-AS1^ promotes CRC cell growth and tumorigenesis in vivo

**Fig. 7:**
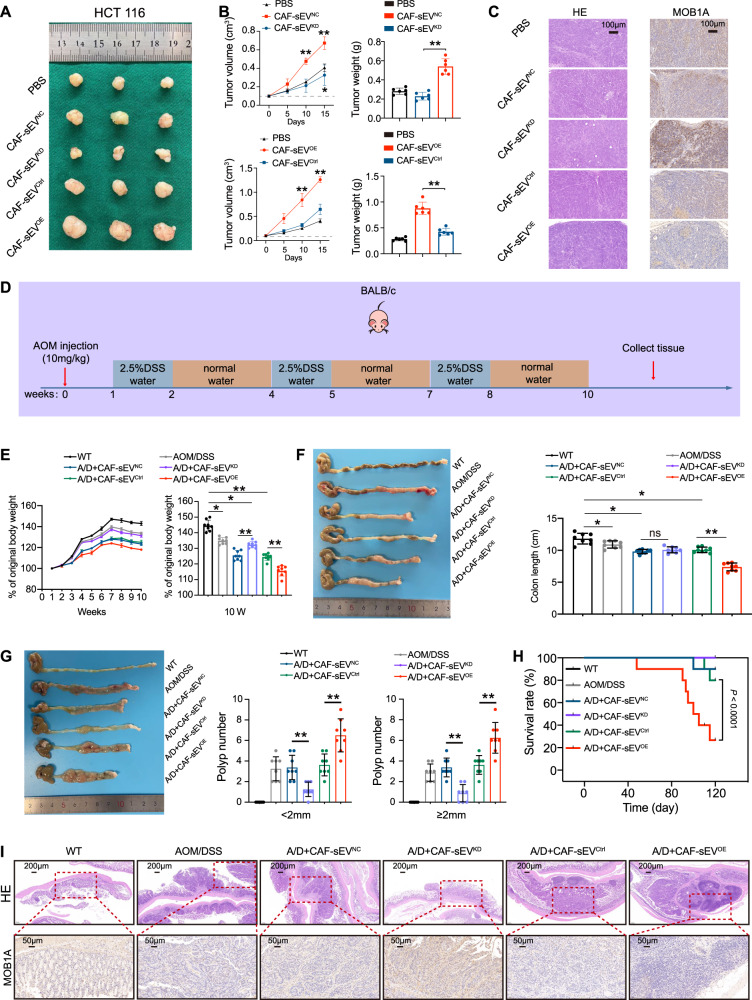
CAF-sEVs^WEE2-AS1^ promote CRC cell growth and tumorigenesis in vivo. **A**, **B** Xenograft tumors of nude mice of different groups with sEVs treatment (*n* = 6 per group). Tumor weight and body weight were measured at the endpoint. **C** HE and IHC of MOB1A in tumors of different groups (scale bar: 100 μm). **D** Schematic diagram of AOM/DSS -induced CRC in BALB/c mice. **E** The relative body weight changes of BALB/c mice are shown with time. **F** Representative images of colons (left) and colon lengths of the WT, AOM/DSS, A/D + CAF-sEVs^NC^, A/D + CAF-sEVs^KD^, A/D + CAF-sEVs^Ctrl^, and A/D + CAF-sEVs^OE^ mice (right), (*n* = 8 per group). **G** Representative images of colons (left) and the number of colonic tumors at least 2 mm or smaller than 2 mm per mouse (right). **H** Survival curves for the indicated mice (*n* = 10 per group). Statistical significance was determined by the Kaplan–Meier log-rank test. **I** HE and IHC of MOB1A in tumors of different groups (scale bar: 200 μm). *P < 0.05, **P < 0.01, ***P < 0.001, ns. not significant.

A subcutaneous xenograft model was established in BALB/c nude mice. When the tumor volume reached 100 mm^3^, we isolated sEVs﻿ from different groups and injected 30 μg sEVs in 50 μl PBS into each group of tumors every five days. As shown in Fig. 7A, B, tumor growth was suppressed by CAF-sEVs^KD^, with lesser tumor volume and weight than control groups. Conversely, CAF-sEVs^OE^ accelerated tumor growth with larger volume and weight compared to their control groups. By performing HE and IHC analysis on mice tumors, we found that MOB1A level was increased in the CAF-sEVs^KD^ group compared to the CAF-sEVs^NC^ group; Contrarily, MOB1A expression was decreased in the CAF-sEVs^OE^ group compared to the CAF-sEVs^Ctrl^ group (Fig. 7C). To verify the effect of sEVs WEE2-AS1 on CRC tumorigenesis, we evaluated AOM/DSS-induced colon cancer (CAC) model in BALB/c mice (Fig. 7D). During CAC induction, exogenous CAF-sEVs were injected into CAC mice via tail vein. As shown in Fig. 7E–G, CAF-sEVs^OE^ accelerated the progression of CAC, as indicated by less weight gain, more colon shortening, more tumor nodules, and more serious colorectal tissue damage. Conversely, CAF-sEVs^KD^ abrogated susceptibility to CAC. More importantly, the CAF-sEVs^OE^ group had a lower survival rate compared to the control group (Fig. 7H). By performing HE and IHC analysis on mice colorectal tissues, we found that MOB1A level was increased in the CAF-sEVs^KD^ group compared to the CAF-sEVs^NC^ group, while MOB1A expression was decreased in the CAF-sEVs^OE^ group compared to the CAF-sEVs^Ctrl^ group (Fig. 7I). Collectively, these findings provided genetic evidence supporting our hypothesis that CAF-sEVs^WEE2-AS1^ promotes CRC cell growth and AOM/DSS-induced tumorigenesis in mice.

### Upregulated WEE2-AS1 in plasma sEVs correlates with poor prognosis of CRC patients

**Fig. 8:**
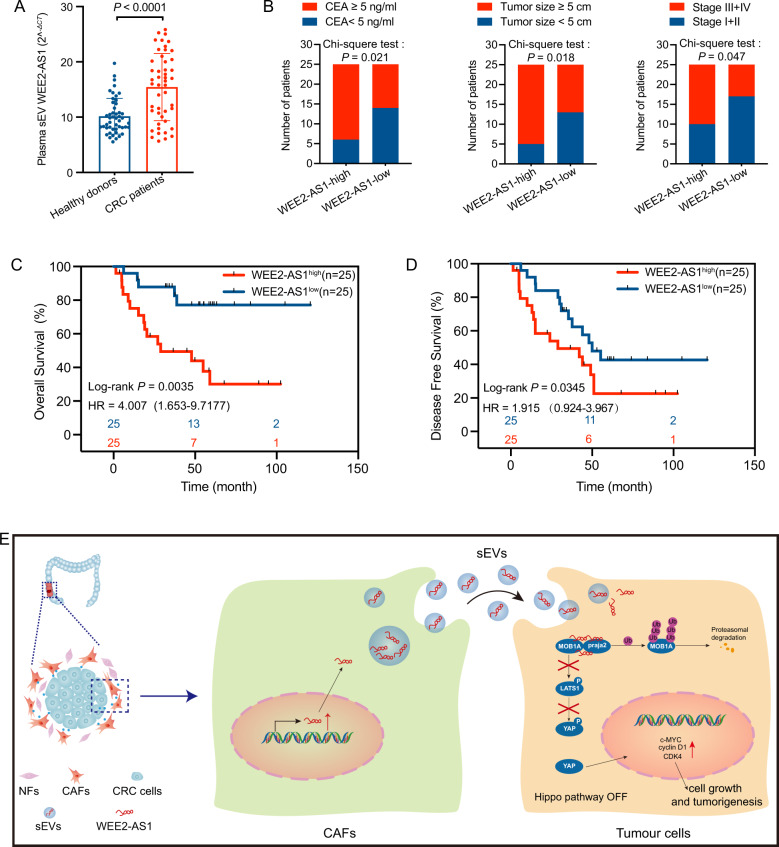
Upregulated plasma sEVs WEE2-AS1 correlates with poor prognosis of CRC patients. **A** Expression of plasma sEVs WEE2-AS1 in healthy donors (*n* = 50) and CRC patients (*n* = 50). **B** High plasma sEVs WEE2-AS expression was correlated with increased CEA level, tumor size, and TNM stage in 50 CRC patients. **C**, **D**. Kaplan–Meier overall survival and disease-free survival curves according to WEE2-AS1 expression levels. **E** Concise model of CAF-sEVs^WEE2-AS1^ in regulating CRC progression.

We isolated and detected the expression of plasma sEVs WEE2-AS1 of 50 CRC patients plasma samples and 50 healthy subjects as described above. As shown in Fig. 8A, plasma sEVs WEE2-AS1 was increased compared with the control. Furthermore, we divided all patients into two groups by the median value (n = 25 > median; n = 25 < median). Clinical correlation analysis revealed that the expression of sEVs WEE2-AS1 was associated with CEA, tumor size, and TNM stage (Fig. 8B, Table S1). Moreover, Kaplan–Meier analysis showed higher sEVs WEE2-AS1 predicted poorer overall survival and disease-free survival than lower group (Figs. 8C, D). Overall, these findings suggest that WEE2-AS1 enriched in sEVs was related to poor prognosis and might be a promising biomarker for CRC.

## Discussion

Causations for initiation and progression of CRC are complicated and may include multiple complex oncogenic alterations, such as environmental, genetic, and epigenetic factors [2]. Understanding the potential mechanisms, and exploring effective treatment strategies against cancer progression are highly desired in the clinic. The emerging evidence reveals that the paradigms of cancer-centric therapeutics have limited therapeutic options in the clinic. TME is the “soil” for tumor survival and provides a place for communication between cancer cells and others. Focusing on the other abundant cellular components in TME, such as fibroblasts and macrophages, has provided a new idea for the study of tumor tumorigenesis and development. Recently, CAFs have received increasing attention due to their crucial roles in the TME. In our study, we confirmed that CAF-sEVs promoted CRC cells growth. The oncogenic effect of CAF-sEVs was also consistent with the previous relevant researches [16]. Then, we re-analyzed the previous sequencing data on CAF-sEVs and screened differential expressed non-coding RNAs. Through the validation of population samples, we identified an oncogenic lncRNA, WEE2-AS1, which was upregulated in CAF-sEVs compared with NF-sEVs.

WEE2-AS1 has been frequently considered as a classical oncogene and is involved in proliferation, apoptosis, and metastasis in various cancer. For example, WEE2-AS1 promotes breast cancer cells proliferation and inhibits apoptosis [29]. In hepatocellular carcinoma, WEE2-AS1 accelerates the proliferation, migration of HCC cells [30]. In addition, WEE2-AS1 not only has a pro-oncogenic effect on tumor cells but also regulates human vascular endothelial cell viability [31]. However, so far, the role of WEE2-AS1 in CRC has not been elucidated. Our study revealed that WEE2-AS1 in CAFs could be secreted into TME via sEVs. Unsurprisingly, we found that CAF-sEVs^WEE2-AS1^ promoted CRC cell proliferation, and inhibited cell apoptosis in vitro. Furthermore, the subcutaneous xenograft model also showed that CAF-sEVs^WEE2-AS1^ promoted CRC cell growth in vivo. Importantly, we established AOM/DSS-induced cancer (CAC) models and found that CAF-sEVs^WEE2-AS1^ promoted more and larger colorectal tumor formation in AOM/DSS-induced CRC. Together, our results indicate that WEE2-AS1 can act as an oncogenic lncRNA in CRC.

The hippo signaling pathway has crucial roles in the control of organ size, carcinogenesis, tissue regeneration, and dysregulation of the Hippo pathway leads to uncontrolled growth and malignant transformation [32, 33]. MOB1A is the core kinase component of Hippo signaling and activated MOB1A promotes the activation of LATS1 and LATS2. Then, activated LATS1 and LATS2, in turn, phosphorylate YAP1 and TAZ, leading to the retention of YAP1 and TAZ in the cytoplasm [33–35]. The depletion of MOB1A leads to the termination of the kinase phosphorylation cascade. Then, stabilized YAP/TAZ complex will translocate to the nucleus and cause transcriptional activation of oncogenes [33]. In our study, WEE2-AS1 endocytosed by CRC cells was mainly localized in the cytoplasm. Nevertheless, we confirmed that WEE2-AS1 could not act as a ceRNA for miRNA. Indeed, WEE2-AS1 functioned as a modular scaffold for the MOB1A and praja2 complexes, and accelerated MOB1A proteasomal degradation. Degradation of MOB1A blocked the phosphorylation cascade of LATS1 and YAP. Stabilized YAP translocated to the nucleus, where it cooperated with TAZ to regulate the activity of transcription factors TEAD, leading to expression of target genes such as c-MYC, cyclin D1, and CDK4.

Liquid biopsy refers to the technique of molecular analysis of the genetic phenotype of a disease based on the circulating genetic material in body fluids [36]. Currently, the main targets of liquid biopsy are circulating tumor DNA (ctDNA), circulating tumor cells (CTCs), and exosomes [37]. Exosomes contain large amounts of proteins, along with a large number of mRNAs, miRNAs, and lncRNAs, while exosomes produced by tumor cells can be detected as overexpressed markers in circulation. Recent studies reveal the emerging role of the exosomal biomarker for cancer diagnosis and prognosis assessment. For example, higher serum exosomal miR-25-3p predicts CRC metastasis [38]. Exosomal miR-196a is negatively correlated with overall survival in head and neck cancer [15]. In our studies, we confirmed that plasma sEVs WEE2-AS1 could be detected in CRC patients and healthy subjects, and was upregulated in CRC patients than the control group. Furthermore, overexpression of WEE2-AS1 is associated with Tumor size, TNM stage, and CEA (ng/ml). Kaplan–Meier analysis showed higher plasma sEVs WEE2-AS1 predicted poorer overall survival and disease-free survival than lower group. Therefore, our findings conclude that upregulated plasma sEVs WEE2-AS1 might serve as a potential diagnostic and prognostic biomarkers for CRC.

We are aware that our study has many limitations that should be taken into account. First, we only confirmed that CAF-sEVs^WEE2-AS1^ promoted CRC proliferation, however, whether it could play a crucial role in CRC metastasis or other malignant phenotypes is unclear. Secondly, it is worth exploring whether CRC cells can mediate the activation of fibroblasts and thus contribute to the malignant transformation of fibroblasts. Thirdly, it warrants further investigation of the mechanism that WEE2-AS1 is high expressed in CAFs than in NFs. There are still many problems worth exploring and studying and we will further explore these aforementioned questions in subsequent experiments.

In summary, our results indicated CAF-secreted sEVs WEE2-AS1 interacts with its protein partner MOB1A and praja2 to function as a modular scaffold and promote proteasomal degradation of MOB1A, inhibit the Hippo pathway, and facilitate the growth of CRC cells. More importantly, increased plasma sEVs WEE2-AS1 might be a meaningful circulating biomarker for CRC diagnosis and prognosis. These findings provide a solid evidence base for a novel therapeutic target.

## Meterials and methods

### Human specimens and cell lines

All samples were collected from CRC patients in the First Affiliated Hospital of Nanjing Medical University. Their blood samples were also collected before resection on the day of surgery. Besides, a total of 50 cases of human blood samples were obtained from healthy donors. All subjects signed informed consent. The ethics permission number is 2019-SRFA-131.

Cell lines (HCT 116, HT-29, and HEK293T) were purchased from the Shanghai Institute of Cell Biology and cultured in McCoy’5A and RPMI-1640 medium (HyClone, Logan) supplemented with 10% FBS.

### Isolation and culture of fibroblasts

CAFs and NFs were isolated from tumor tissues, paired normal tissues (at least 5 cm from the outer tumor margin), respectively. In brief, the fresh paired tissues were washed with PBS including 100 U/ ml penicillin and 100 μg/ml streptomycin three times. Then, the tissues were cut and digested in collagenase II (Gibco, USA) for one hour. All cells were collected and resuspended in DMEM/F12 medium. Then the single-cell suspensions were added to the culture dishes for 1 h, allowing fibroblasts to attach to the plates. Unattached cells were removed after one hour of incubation. The fibroblasts-specific markers (α-SMA, S100A4, and Vimentin) were used to identify the fibroblasts. All primary fibroblasts used in this study were less than ten passages.

### Small extracellular vesicles isolation and characterization

Fibroblasts were cultured in the complete medium until growing to 60%. After twice-washing in PBS, the complete medium was replaced by a medium with 10% exosomes-depleted FBS. After 2 days, the cellular supernatant was collected and centrifuged at 500 *g* for 5 min, 2000 *g* for 15 min, 12,000 *g* for 30 min to remove the dead cells and cellular debris. Then, the supernatant was ultracentrifuged (BeckmanCoulter Avanti J-30I, USA) at 120,000 *g* for 70 min and the pellet was suspended in ice-cold PBS before ultracentrifuged at 120,000 *g* for 70 min again. Plasma sEVs were isolated using the ExoQuick kit (Qiagen, Frederick, MD, USA). Before being used, sEVs were filtrated through 0.22μm filters.

Nanoparticle tracking analysis (NTA) was used to examine the diameter and concentration of sEVs. The morphology of sEVs was analyzed by ﻿transmission electron microscopy (TEM). CD63, CD81, and TSG101 were used for sEVs markers while Calnexin was used as a negative control for sEVs.

### sEVs labeling and tracing

The isolated sEVs were labeled by PKH67 (Sigma-Aldrich, USA) and then the labeled sEVs were added to the supernatant. After incubation with recipient cells for 24 h, cells were observed by confocal microscopy (Leica, Germany). The nuclei were stained with DAPI.

### Western Blot (WB) analysis and antibodies

Cells or sEVs were cracked with lysis buffer as reported previously [39]. The primary antibodies are listed in Table S2.

### RNA extraction and qRT-PCR

The total RNA was isolated as reported previously [39]. The sEVs RNA was extracted from plasma and culture medium using the ExoQuick kit (Umibio, Shanghai).

For qRT-PCR of mRNA, total RNA was reverse transcribed into cDNA through the RT Mix (Vazyme, Jiangsu, China). For miRNA RT-PCR, the Reverse Transcription Kit (RiboBio, Guangzhou, China) was used to perform target-specific reverse transcription. For cell and sEVs samples, the expression level was normalized to internal controls (GAPDH) and external controls cel-miR-39 (RiboBio), respectively. The primer sequences are listed in Table S3 and the results are calculated by the 2^−ΔCT^ method.

### RNA and protein isolation of nuclear and cytoplasmic fractions

PARIS kit (#AM1921; ThermoFisher) was used to separate cytoplasmic and nuclear fractions as reported previously [39]. The levels of WEE2-AS1 expression were analyzed using qRT-PCR. For qRT-PCR, U6 and GAPDH were used as a nuclear or cytoplasmic reference, respectively. For WB, YY1 and GAPDH were used as nuclear or cytoplasmic references.

### RNA interference and plasmids

The lentivirus (LV) containing shRNAs targeting WEE2-AS1 were designed and synthesized by Genomeditech (Shanghai, China). The full length of WEE2-AS1 synthesized by Genomeditech was subcloned into lentivirus vector.

shRNAs (three shWEE2-AS1 and shNC) plasmids, WEE2-AS1 plasmids, Ub-HA-tagged plasmids, MOB1A-Flag-tagged plasmids, praja2-His-tagged plasmids were designed and synthesized by Genomeditech (Shanghai, China). The sequences are listed in Table S3.

### Cell proliferation assay

Cells were co-cultured with sEVs for 24 h before the next study. CCK-8 assay and colony formation assay were performed as described previously [39].

### Cell cycle and apoptosis analysis

Cells were co-cultured with sEVs for 24 h before the next study. Then, the cells were treated with cisplatin (5ug/ml for 24 h) [39, 40]. The percentages of cells in the G0/G1, S, and G2/M phases were analyzed by BD FACSCanto II (BD Biosciences, USA). The apoptotic rate was analyzed using BD FACSCanto II.

### RNA-protein immunoprecipitation (RIP)

A Magna RIP Kit (Millipore, USA) was used as reported previously [39]. 50 µL of a magnetic beads suspension was washed and resuspended in 100 µL of the wash buffer. Then, antibodies were added into each tube to form a beads-antibody complex. Next, 100 µL lysates were added to each beads-antibody complex and all the tubes were incubated while rotating overnight at 4 °C. The purified RNA was analyzed by qRT-PCR.

### Fluorescence in situ hybridization

A FISH Kit (RiboBio) was applied to detect the location of lncRNA as reported previously [39]. Results were observed by confocal microscopy.

### Immunofluorescence (IF)

Cells were fixed with immunostaining fixative (P0098; Beyotime) overnight at 4 °C. Next, cells were blocked with blocking buffer (P0102; Beyotime) and then incubated with primary antibody overnight at 4 °C. The cells were incubated with an Alexa Fluor 488-labeled goat anti-rabbit IgG (A0423, Beyotime) for 60 min in the dark. DAPI was used to visualize the nuclei.

### Luciferase reporter assay

Luciferase construct containing the 3ʹ-UTR of WEE2-AS1 mRNA was synthesized by Genomeditech (Shanghai, China). Cells were transfected with miR-579-3p, miR-6833-3p, miR-644b-3p, miR-4303, and miR-3928-5p miRNA-mimics or miR-NC, respectively. Luciferase activity was measured by Luciferase Reporter Assay System (Promega, USA).

### RNA pulldown assay

WEE2-AS1 probes were synthesized by RiboBio (Guangzhou, China). Cell lysates were incubated with a probe-bead complex for 60 min. The complex was eluted in elution buffer and the resolved protein was used for WB, silver staining, or mass spectrometry analysis (BGI Shenzhen, Guangdong, China).

### Immunohistochemistry

IHC was performed as previously described [39].

### Animal models

BALB/c mice and BALB/c nude mice (male, 6 weeks old) were purchased from the animal center of Nanjing Medical University. The ethical number of the animal experiment is IACUC-2111040.

For the subcutaneous tumor model, 30 nude mice were divided randomly into five groups: PBS, CAF-sEVs ^NC^, CAF-sEVs ^KD^, CAF-sEVs ^Ctrl^, CAF-sEVs ^OE^. First, we injected wild-type HCT 116 cells (4 × 10^6^/150 μl) subcutaneously into the right flank. When tumors grew to 100 mm^3^, we isolated sEVs﻿ from CAF-WEE2-AS1^NC^, CAF-WEE2-AS1^KD^, CAF- WEE2-AS1^Ctrl^, CAF- WEE2-AS1^OE^ groups and injected sEVs (30 μg) in 50 μl PBS into the tumor of each group every five days. Tumors were measured by Vernier calipers every five days and ﻿calculated with the following formula: tumor volume = (length × width^2^) × 0.52. After 15 days, the mice were killed and tumors were dissected and weighed.

For the AOM/DSS model of colorectal tumorigenesis, BALB/c mice were injected intraperitoneally with 10 mg of azoxymethane (AOM, A5486, Sigma) per kg body weight. Starting 1 week after the injection, 2.5% dextran sulfate sodium (DSS, MP Biologicals) was given in the drinking water for 1 week followed by regular drinking normal water for 2 weeks. A cycle is 21 days. This cycle was repeated twice. During this process, the mice were treated with 100 μl PBS, 30 µg CAF-sEVs ^NC^, 30 µg CAF-sEVs ^KD^, 30 µg CAF-sEVs ^OE^, 30 µg CAF-sEVs ^Ctrl^ in 100 μl PBS via tail vein once every week from the first cycle. The animals were constantly monitored for body weight. Mice were sacrificed at the end of the study by bloodletting followed by cervical dislocation and their colon was resected for H&E staining, and IHC assays.

### Statistics analysis

Each experiment was independently performed in triplicate. The data are shown as the mean ± standard deviation. GraphPad Prism 8.0 (GraphPad, La Jolla, CA, USA) was used for the statistical analyses.

## Supplementary information

Figure S1

Figure S2

Figure S3

Figure S4

Figure S5

Table S1

Table S2

Table S3

Table S4

Supplementary figure legends

Original Data File

aj-checklist

## Data Availability

The datasets used in the current study are available from the corresponding author on reasonable request.

## Code availability

Codes are available upon reasonable request.
