# Supplementary figures and images for: CAF-derived exosomal WEE2-AS1 facilitates colorectal cancer progression via promoting degradation of MOB1A to inhibit the Hippo pathway
Source: Cell Death Dis. 2022 Sep 19;13(9):796. doi: 10.1038/s41419-022-05240-7 (PMC9485119; doi:10.1038/s41419-022-05240-7)

**A**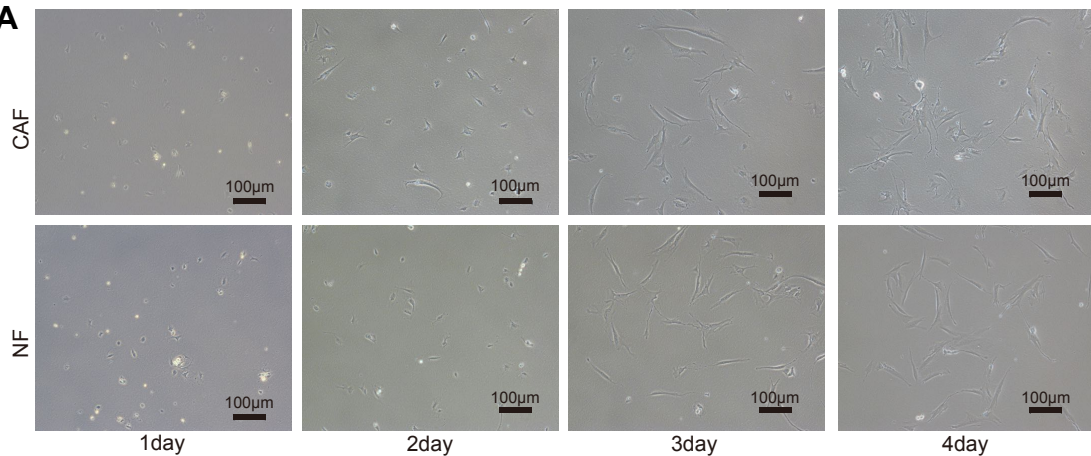**B**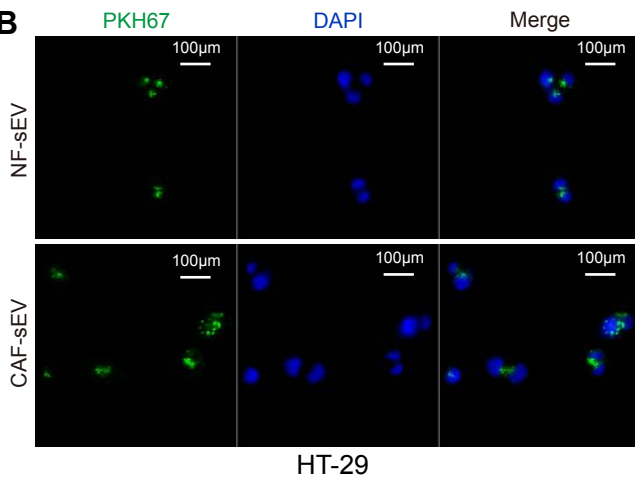**C**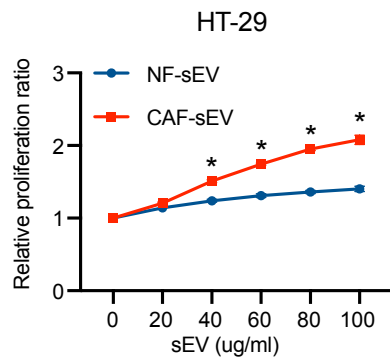

Supplement: Supplementary file 1 — Figure S1 [file 41419_2022_5240_MOESM1_ESM.pdf]

A

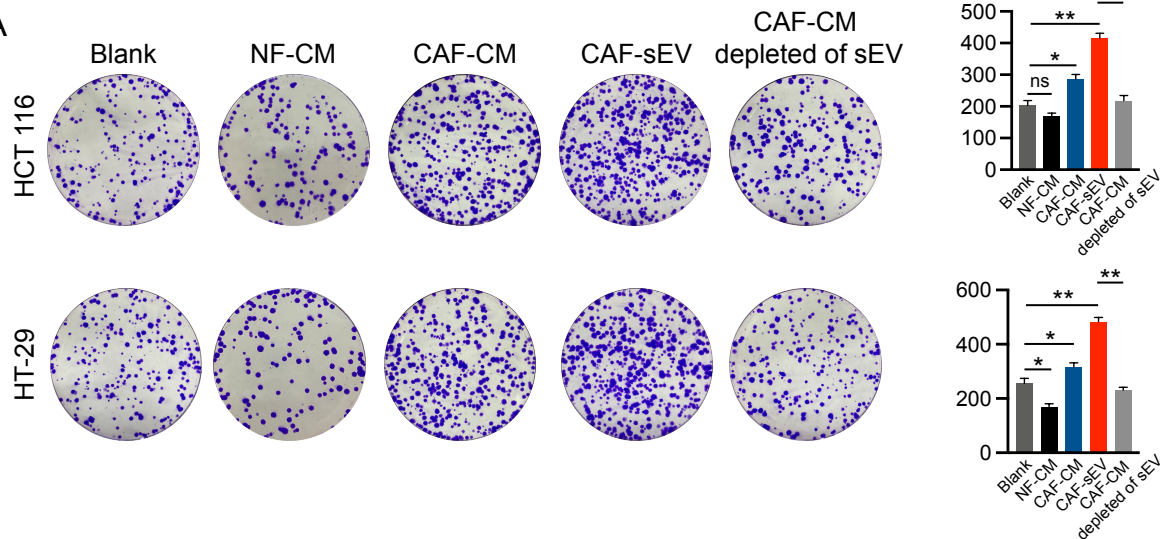

B

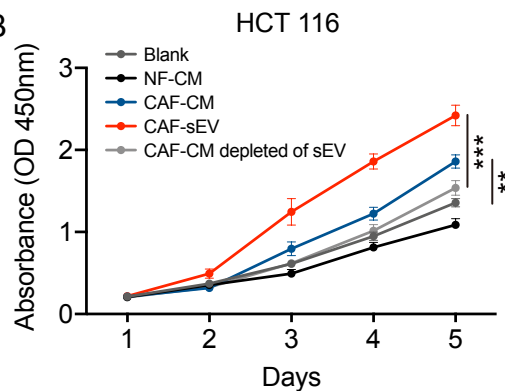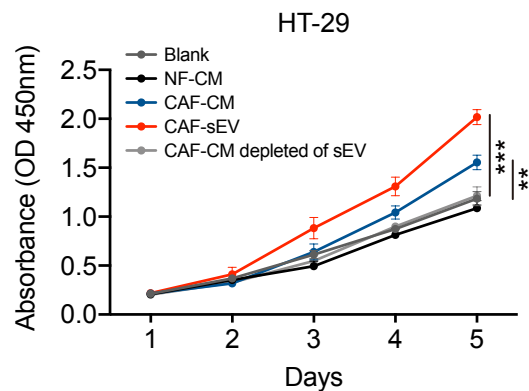

Supplement: Supplementary file 2 — Figure S2 [file 41419_2022_5240_MOESM2_ESM.pdf]

**A**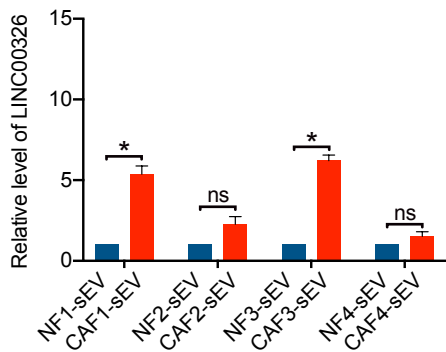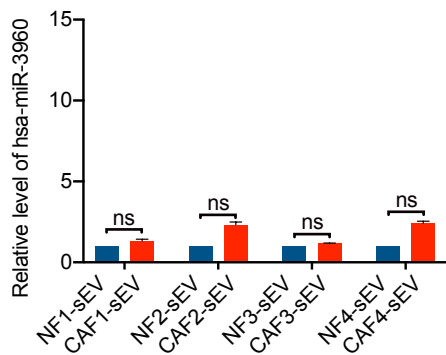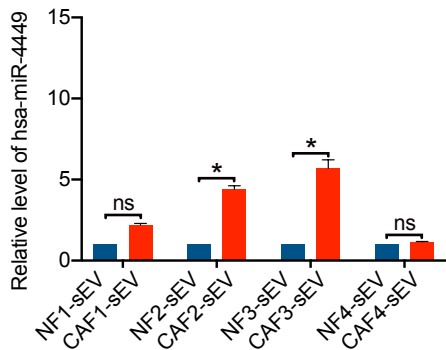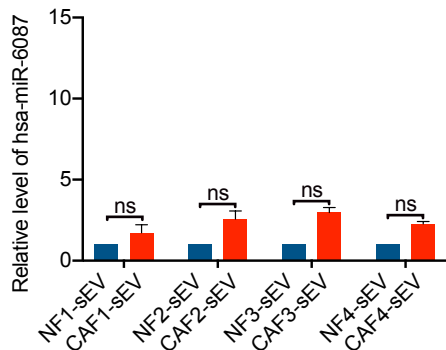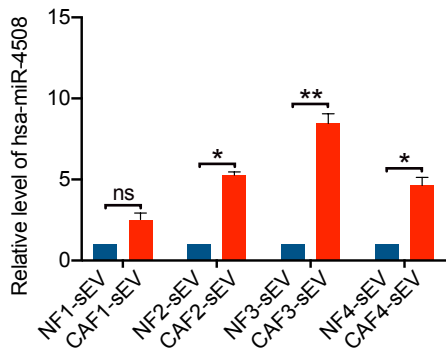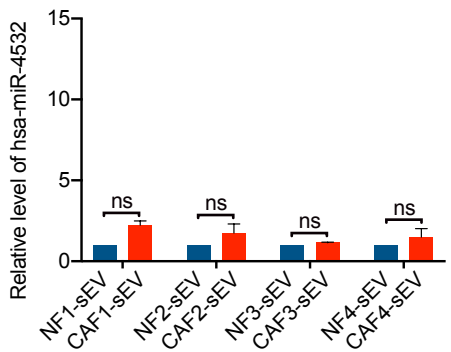

Supplement: Supplementary file 3 — Figure S3 [file 41419_2022_5240_MOESM3_ESM.pdf]

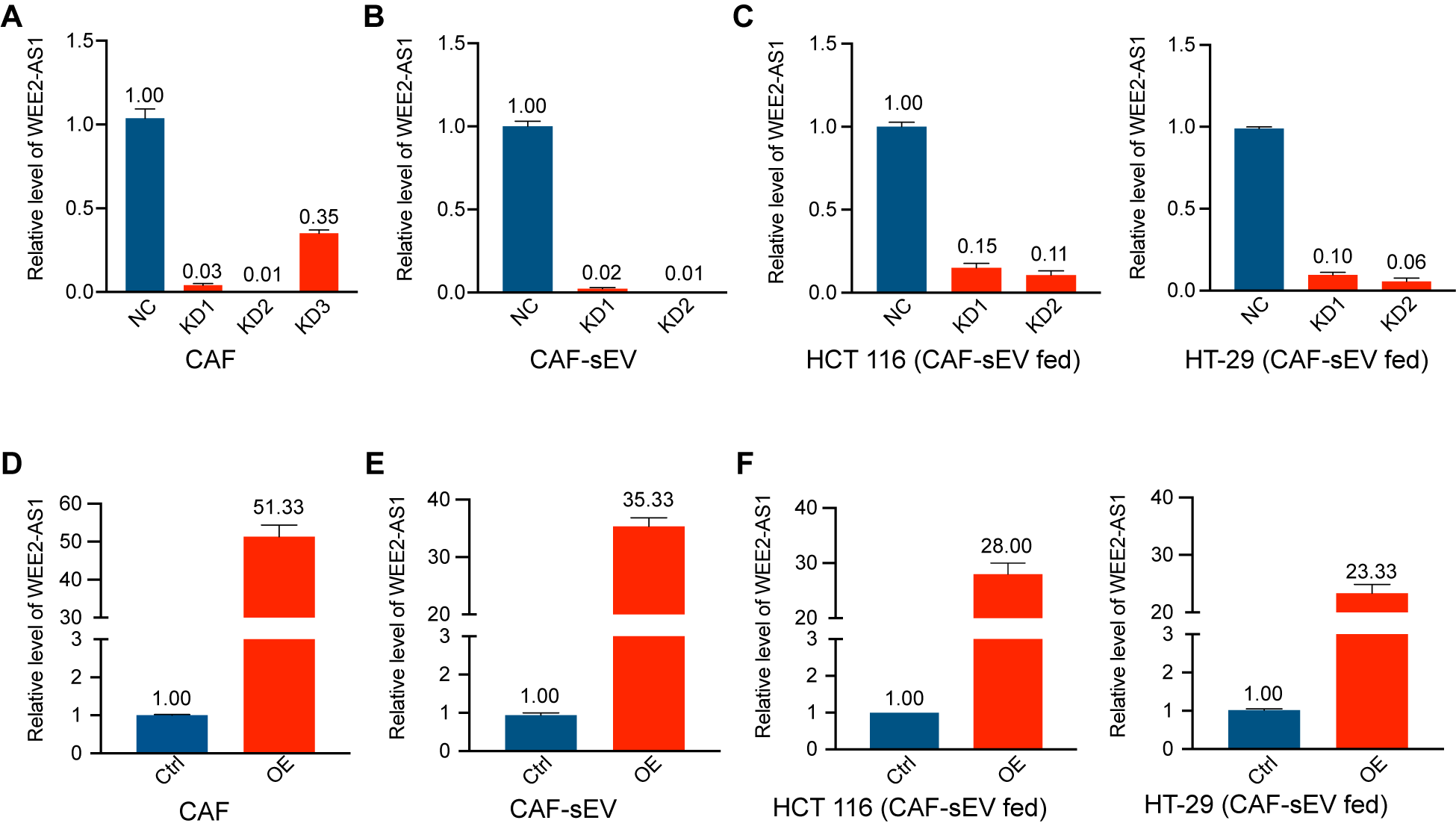

Supplement: Supplementary file 4 — Figure S4 [file 41419_2022_5240_MOESM4_ESM.tif]

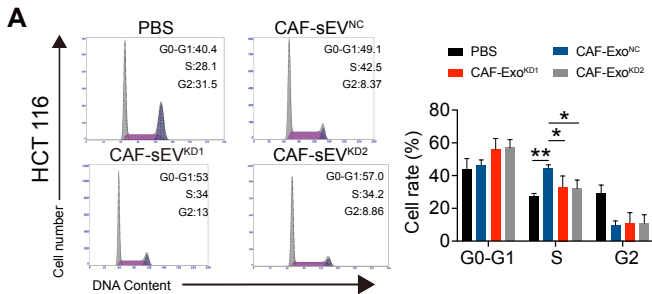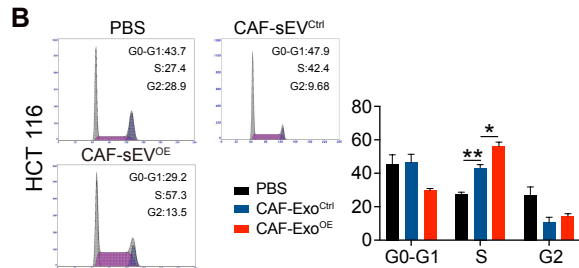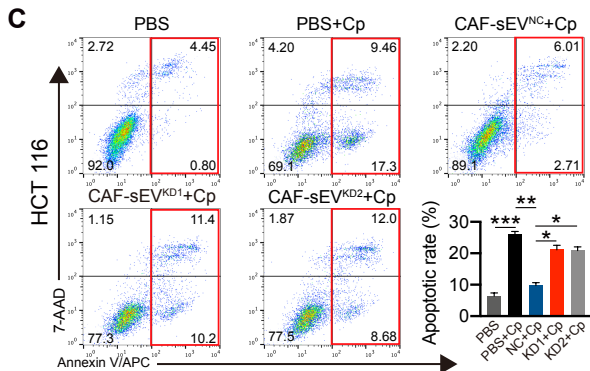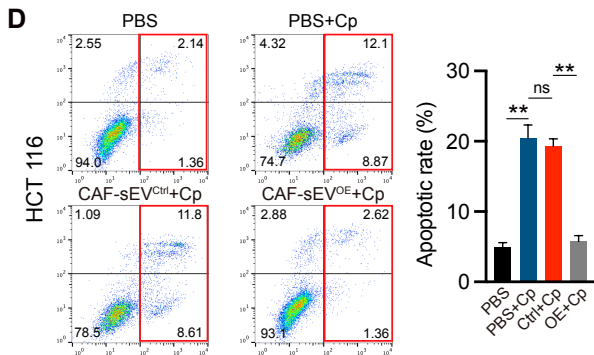

Supplement: Supplementary file 5 — Figure S5 [file 41419_2022_5240_MOESM5_ESM.pdf]

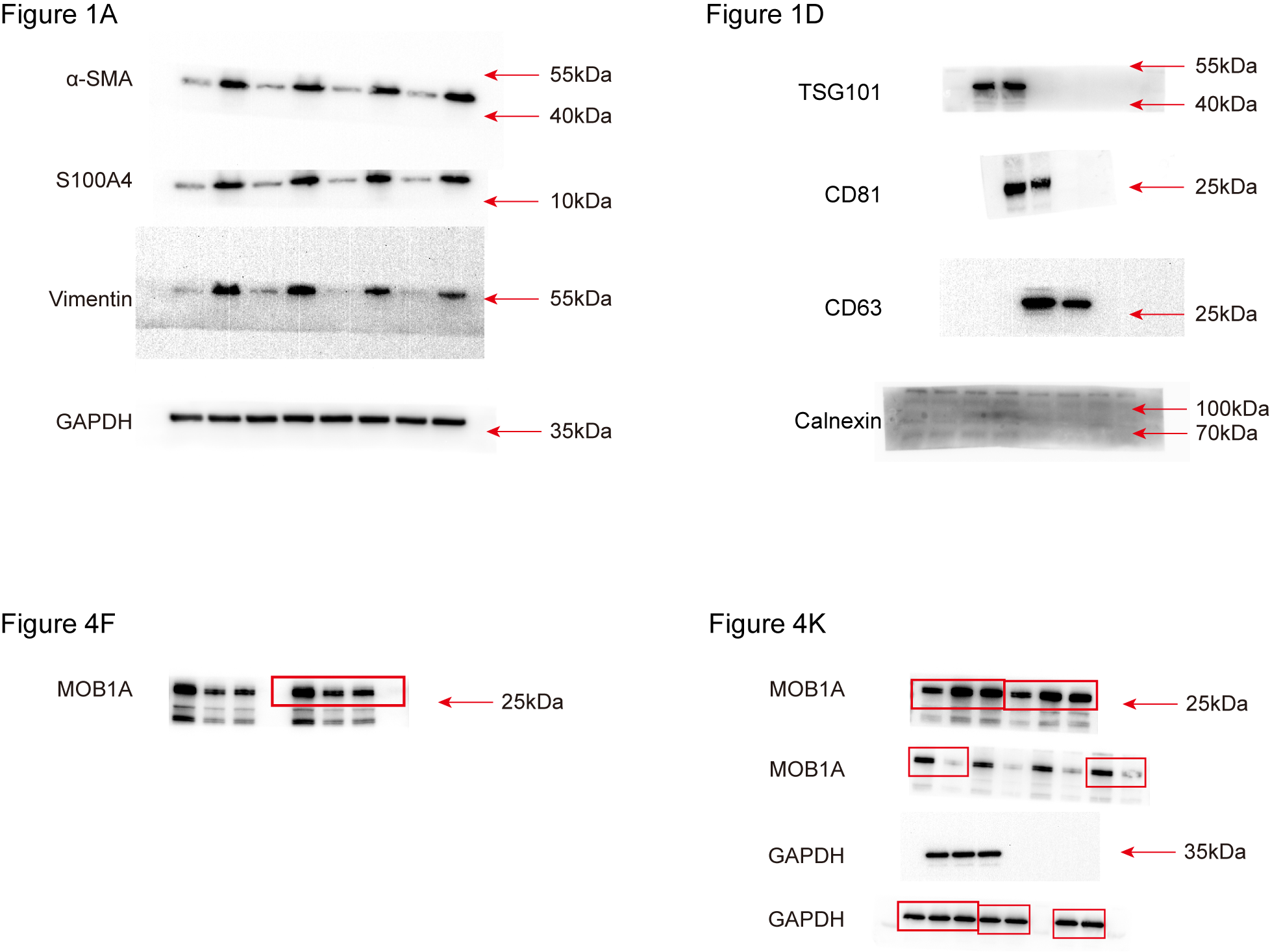

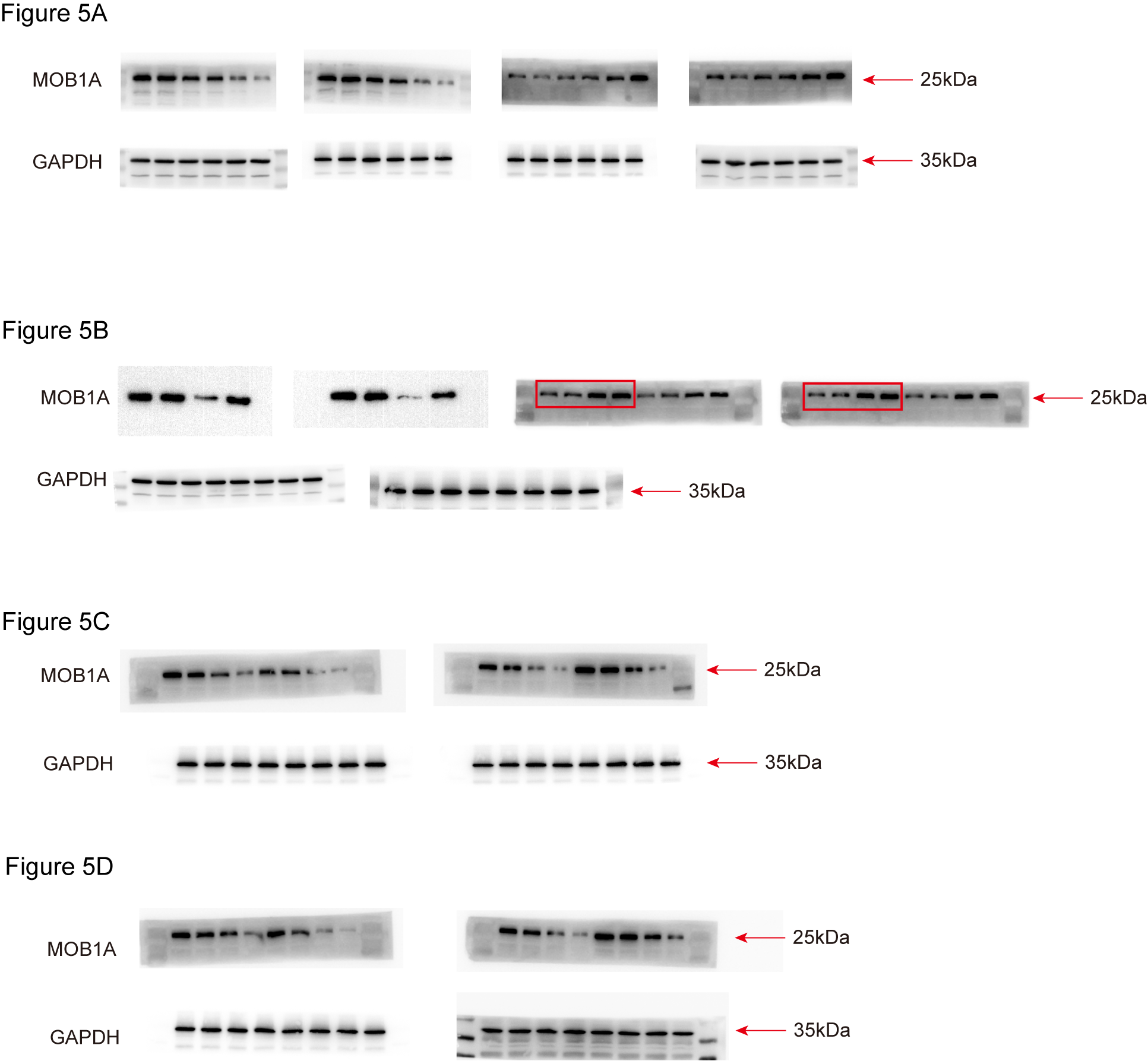

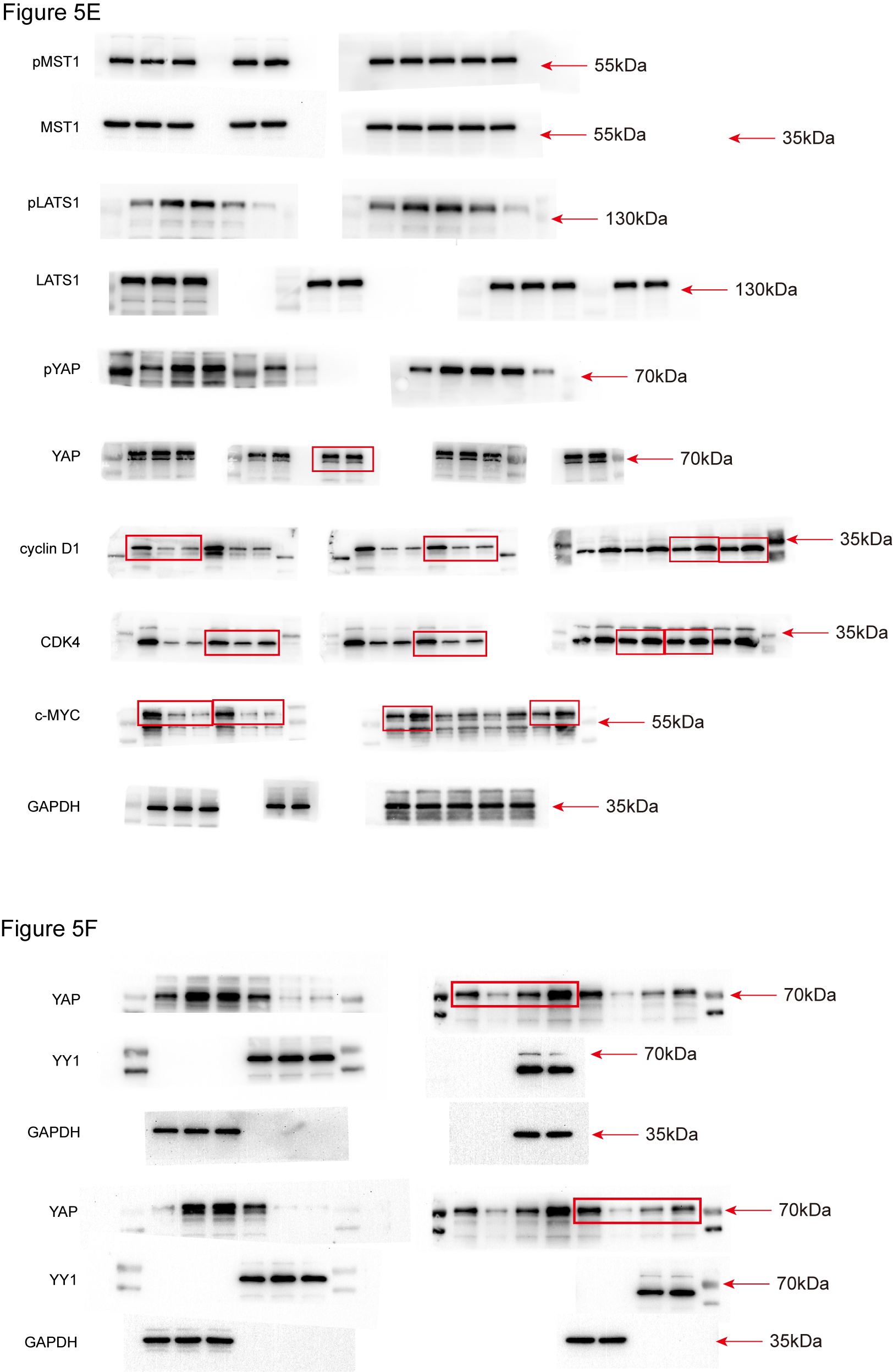

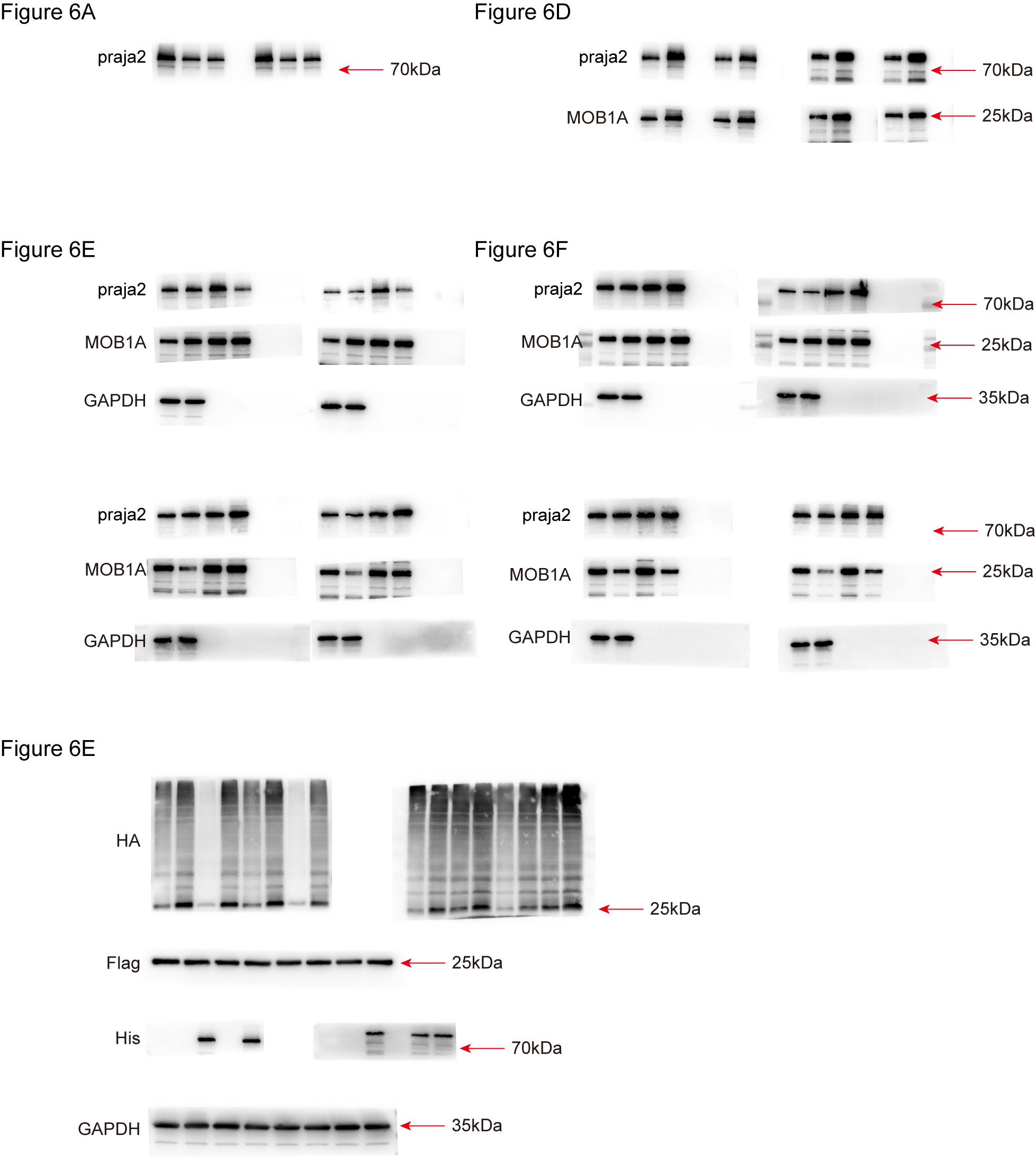

Supplement: Supplementary file 11 — Original Data File [file 41419_2022_5240_MOESM11_ESM.docx]
