# Supplementary material for: CAF-derived exosomal WEE2-AS1 facilitates colorectal cancer progression via promoting degradation of MOB1A to inhibit the Hippo pathway
Source: Cell Death Dis. 2022 Sep 19;13(9):796. doi: 10.1038/s41419-022-05240-7 (PMC9485119; doi:10.1038/s41419-022-05240-7)
Supplement: Supplementary file 6 — Table S1 [file 41419_2022_5240_MOESM6_ESM.docx]

| **Table S1: Relevance analysis of sEV WEE2-AS1 expression in CRC patients.** | | | | |
| --- | --- | --- | --- | --- |
| **Variable** | **All patients** | **sEV WEE2-AS1** | | **P-value** |
|  |  | **High** | **Low** |  |
| All Cases | 50 | 25 | 25 |  |
| Age (years) |  |  |  |  |
| <60 | 17 | 9 | 8 | 0.765 |
| ≥60 | 33 | 16 | 17 |  |
| Gender |  |  |  |  |
| Male | 31 | 17 | 14 | 0.382 |
| Female | 19 | 8 | 11 |  |
| Tumor size (cm) |  |  |  |  |
| <5 | 18 | 5 | 13 | **0.018** |
| ≥5 | 32 | 20 | 12 |  |
| TNM staging system |  |  |  |  |
| Stage I+II | 27 | 10 | 17 | **0.047** |
| Stage III+IV | 23 | 15 | 8 |  |
| Lymph node metastasis |  |  |  |  |
| No | 29 | 12 | 17 | 0.152 |
| Yes | 21 | 13 | 8 |  |
| CEA (ng/ml) |  |  |  |  |
| <5 | 20 | 6 | 14 | **0.021** |
| ≥5 | 30 | 19 | 11 |  |

NOTE: TNM tumor node metastasis. CEA carcinoembryonic antigen

P ≤ 0.05 was considered signiﬁcant. The bold type represents P-values smaller than 0.05
