# Supplementary material for: CAF-derived exosomal WEE2-AS1 facilitates colorectal cancer progression via promoting degradation of MOB1A to inhibit the Hippo pathway
Source: Cell Death Dis. 2022 Sep 19;13(9):796. doi: 10.1038/s41419-022-05240-7 (PMC9485119; doi:10.1038/s41419-022-05240-7)
Supplement: Supplementary file 7 — Table S2 [file 41419_2022_5240_MOESM7_ESM.docx]

| **Table S2** | | |
| --- | --- | --- |
| **Antigens** | **Manufacturer** | **Application** |
| α-SMA | ProteinTech : 14395-1-AP | 1:1000 for WB; 1:100 for IF |
| S100A4 | ProteinTech : 16105-1-AP | 1:1000 for WB; 1:100 for IF |
| Vimentin | ProteinTech : 10366-1-AP | 1:1000 for WB; 1:100 for IF |
| CD81 | Abcam : ab109201 | 1:1000 for WB |
| TSG101 | Abcam : ab125011 | 1:1000 for WB |
| CD63 | Abcam : ab134045 | 1:1000 for WB |
| Calnexin | Abcam : ab22595 | 1:1000 for WB |
| Ago2 | Abcam : ab186733 |  |
| MOB1A | Affinity : DF3333 | 1:1000 for WB; 1:100 for IF |
| pMOB1A | Affinity : AF4481 | 1:1000 for WB |
| pMST1 | ProteinTech : 28953-1-AP | 1:1000 for WB |
| MST1 | ProteinTech : 22245-1-AP | 1:1000 for WB |
| LATS1 | Cell Signaling Technology : #3477 | 1:1000 for WB |
| pLATS1 | Cell Signaling Technology : #9157 | 1:1000 for WB |
| YAP1 | Abcam : ab52771 | 1:5000 for WB |
| pYAP1 | Abcam : ab76252 | 1:5000 for WB |
| C-myc | Abcam : ab32072 | 1:1000 for WB |
| Cyclin D1 | Abcam : ab134175 | 1:5000 for WB |
| CDK4 | Abcam : ab108357 | 1:1000 for WB |
| YY1 | Cell Signaling Technology : #63227 | 1:1000 for WB |
| GAPDH | Abcam : ab9485 | 1:2500 for WB |
| praja2 | Affinity :DF4021 | 1:1000 for WB; 1:100 for IF |
| HA | ProteinTech : 51064-2-AP | 1:3000 for WB |
