# Supplementary material for: CAF-derived exosomal WEE2-AS1 facilitates colorectal cancer progression via promoting degradation of MOB1A to inhibit the Hippo pathway
Source: Cell Death Dis. 2022 Sep 19;13(9):796. doi: 10.1038/s41419-022-05240-7 (PMC9485119; doi:10.1038/s41419-022-05240-7)
Supplement: Supplementary file 8 — Table S3 [file 41419_2022_5240_MOESM8_ESM.docx]

**Table S3** Sequences of primers and shRNAs.

| **Table S3** | |
| --- | --- |
| **qRT-PCR Primers** |  |
| WEE2-AS1 | F:5'-CCCAAGCAAGAAATGAC-3' |
|  | R:5'-GAGACCCAGAGAGCCAC-3' |
| GAPDH | F:5'-GGAGCGAGATCCCTCCAAAAT-3' |
|  | R:5'-GGCTGTTGTCATACTTCTCATGG-3' |
| U6 | F:5'-CTCGCTTCGGCAGCACA-3' |
|  | R:5'-AACGCTTCACGAATTTGCGT-3' |
| LINC00326 | F:5'-CGAGCCATCAAGAAGCTTGC-3' |
|  | R:5'-GGGTTTTACTGTGGGGCTGA-3' |
| hsa-miR-3960 | RT:5'-CTCAACTGGTGTCGTGGAGTCGGCAATTCAGTTGAGCCGCCGCC-3' |
|  | F:5'-GCCGACGGGGGCGGAGGCGGCGGCGG-3' |
|  | R:5'-CTCAACTGGTGTCGTGGA-3' |
| hsa-miR-4449 | RT:5'-CTCAACTGGTGTCGTGGAGTCGGCAATTCAGTTGAGGCAGGGCC-3' |
|  | F:5'-GCCGACACGGAGCGCGUCGGGGCCCUGC-3' |
|  | R:5'-CTCAACTGGTGTCGTGGA-3' |
| hsa-miR-6087 | RT:5'-CTCAACTGGTGTCGTGGAGTCGGCAATTCAGTTGAGGCTCGCCC-3' |
|  | F:5'-GCCGACUGAGGCGGGGGGGCGAGC-3' |
|  | R:5'-CTCAACTGGTGTCGTGGA-3' |
| hsa-miR-4508 | RT:5'-CTCAACTGGTGTCGTGGAGTCGGCAATTCAGTTGAGCGCCCCGA-3' |
|  | F:5'-GCCGACGCGCGCGGGUCGGGGCG-3' |
|  | R:5'-CTCAACTGGTGTCGTGGA-3' |
| hsa-miR-4532 | RT:5'-CTCAACTGGTGTCGTGGAGTCGGCAATTCAGTTGAGCGCCGGGC-3' |
|  | F:5'-GCCGACCCCCGGGGAGCCCGGCG-3' |
|  | R:5'-CTCAACTGGTGTCGTGGA-3' |
| **short hairpin RNAs (shRNAs) sequence** |  |
| sh-WEE2-AS1 #1 | sense:5'GATCCGTTCTGATGATATAGGACCAACTCGAGTTGGTCCTATATCATCAGAACTTTTTG-3' |
|  | antisense:5'AATTCAAAAAGTTCTGATGATATAGGACCAACTCGAGTTGGTCCTATATCATCAGAACG-3' |
| sh-WEE2-AS1 #2 | sense:5'GATCCCTTCTATGACTCCAGAGGATTCTCGAGAATCCTCTGGAGTCATAGAAGTTTTTG-3' |
|  | antisense:5'AATTCAAAAACTTCTATGACTCCAGAGGATTCTCGAGAATCCTCTGGAGTCATAGAAGG-3' |
| sh-WEE2-AS1 #3 | sense:5'GATCCCAGAAGCTTCACTATTGGGAACTCGAGTTCCCAATAGTGAAGCTTCTGTTTTTG-3' |
|  | antisense:5'AATTCAAAAACAGAAGCTTCACTATTGGGAACTCGAGTTCCCAATAGTGAAGCTTCTGG-3' |
