# Supplementary material for: CAF-derived exosomal WEE2-AS1 facilitates colorectal cancer progression via promoting degradation of MOB1A to inhibit the Hippo pathway
Source: Cell Death Dis. 2022 Sep 19;13(9):796. doi: 10.1038/s41419-022-05240-7 (PMC9485119; doi:10.1038/s41419-022-05240-7)
Supplement: Supplementary file 9 — Table S4 [file 41419_2022_5240_MOESM9_ESM.docx]

| **Table S4:Mass spectrometry assay revealed 71 differential proteins** | | | |
| --- | --- | --- | --- |
| **Protein names** | **Gene names** | **Protein_ID** | **Score** |
| MOB kinase activator 1A | MOB1A | sp\|Q9H8S9 | 323.31 |
| Plectin | PLEC | sp\|Q15149 | 306.22 |
| Ubiquitin carboxyl-terminal hydrolase 1 | USP1 | sp\|O94782 | 231.02 |
| 40S ribosomal protein S17 | RPS17 | tr\|H3BNC9 | 204.19 |
| E3 ubiquitin-protein ligase praja2 | praja2 | sp\|O43164 | 181.88 |
| Actin, cytoplasmic 2 | ACTG1 | sp\|P63261 | 150.98 |
| Protein FAM83H | FAM83H | tr\|A0A494C1T9 | 125.09 |
| Myosin-9 | MYH9 | tr\|A0A024R1N1 | 123.33 |
| Elongation factor 1-alpha 1 | EEF1A1 | tr\|A0A4D5RBG5 | 105.32 |
| Heat shock 70 kDa protein 6 | HSPA6 | sp\|P17066 | 95.03 |
| Nuclease-sensitive element-binding protein 1 | YBX1 | sp\|P67809 | 83.65 |
| Desmoplakin | DSP | sp\|P15924 | 83.095 |
| Plasminogen activator inhibitor 1 RNA-binding protein | DKFZp686P17171 | tr\|Q63HR1 | 82.579 |
| Fragile X mental retardation syndrome-related protein 1 | FXR1 | tr\|A0A0F7KYT8 | 79.984 |
| Heterogeneous nuclear ribonucleoproteins C1/C2 | HNRNPC | tr\|G3V4W0 | 79.744 |
| Thrombospondin-1 | THBS1 | sp\|P07996 | 79.554 |
| Enhancer of rudimentary homolog | ERH | tr\|A0A024R6D4 | 76.776 |
| Heat shock cognate 71 kDa protein | HEL-S-72p | tr\|V9HW22 | 75.279 |
| Gelsolin | GSN | tr\|B7Z992 | 74.361 |
| 40S ribosomal protein S2 | RPS2 | tr\|Q3KQT6 | 73.694 |
| Heat shock protein HSP 90-alpha | HSP90AA1 | tr\|Q2VPJ6 | 68.762 |
| Filaggrin-2 | FLG2 | sp\|Q5D862 | 64.738 |
| 40S ribosomal protein S8 | RPS8 | tr\|Q5JR95 | 64.462 |
| Protein S100-A7;Protein S100-A7A | S100A7 | sp\|P31151 | 63.947 |
| Nucleophosmin | NPM1 | tr\|A0A0S2Z4G7 | 62.167 |
| Lamina-associated polypeptide 2, isoform alpha | TMPO | sp\|P42166 | 56.789 |
| Periplakin | PPL | sp\|O60437 | 55.424 |
| Programmed cell death protein 2-like | PDCD2L | sp\|Q9BRP1 | 54.802 |
| Protein SEC13 homolog | SEC13 | tr\|Q53GB2 | 54.536 |
| SH3KBP1-binding protein 1 | SHKBP1 | tr\|M0R2P6 | 53.913 |
| HCLS1-associated protein X-1 | HAX1 | tr\|A0A0S2Z591 | 53.585 |
| Desmocollin-1 | DSC1 | tr\|Q9HB00 | 52.531 |
| 40S ribosomal protein S29 | RPS29 | tr\|A0A2R8Y851 | 51.474 |
| Fragile X mental retardation syndrome-related protein 2 | FXR2 | sp\|P51116 | 51.368 |
| Thyroid hormone receptor-associated protein 3 | THRAP3 | sp\|Q9Y2W1 | 50.676 |
| Heterogeneous nuclear ribonucleoprotein H | HNRNPH1 | tr\|E9PCY7 | 50.627 |
| Filaggrin | FLG | tr\|I0B0K8 | 50.226 |
| Ribonucleases P/MRP protein subunit POP1 | POP1 | tr\|Q96F88 | 50.11 |
| Unconventional myosin-Ic | MYO1C | tr\|F5H6E2 | 49.758 |
| X-ray repair cross-complementing protein 6 | XRCC6 | tr\|A0A024R1N4 | 49.567 |
| Tyrosine-protein kinase receptor;Sequestosome-1 | SQSTM1 | tr\|E9PFW8 | 48.922 |
| Heat shock protein beta-1 | HEL-S-102 | tr\|V9HW43 | 48.3 |
| Glyceraldehyde-3-phosphate dehydrogenase | GAPDH | tr\|E7EUT5 | 47.993 |
| Heat shock protein HSP 90-beta | HSP90AB1 | tr\|B4DMA2 | 47.817 |
| Polyadenylate-binding protein | PABPC1 | tr\|E7EQV3 | 47.036 |
| E3 ubiquitin-protein ligase TRIM21 | TRIM21 | sp\|P19474 | 46.014 |
| Heterogeneous nuclear ribonucleoprotein M | HNRNPM | tr\|A0A087X0X3 | 45.732 |
| Splicing factor U2AF 35 kDa subunit | U2AF1 | sp\|P0DN76 | 43.616 |
| Caseinolytic peptidase B protein homolog | CLPB | tr\|A0A140VK11 | 42.895 |
| Protein S100-A14 | S100A14 | sp\|Q9HCY8 | 42.8 |
| Bcl-2-associated transcription factor 1 | BCLAF1 | tr\|E9PK91 | 41.629 |
| BTB/POZ domain-containing protein KCTD3 | KCTD3 | sp\|Q9Y597 | 41.23 |
| Fragile X mental retardation protein 1 | FMR1 | tr\|X5DQX7 | 40.062 |
| Serum albumin | ALB | tr\|F6KPG5 | 39.524 |
| Ubiquitin-60S ribosomal protein L40 | UBB | tr\|J3QS39 | 39.435 |
| Protein-L-isoaspartate O-methyltransferase | PCMT1 | tr\|B7Z972 | 39.323 |
| Heterogeneous nuclear ribonucleoproteins A2/B1 | HNRPA2B1 | tr\|A0A024RA28 | 37.984 |
| Ribonuclease P protein subunit p30 | RPP30 | sp\|P78346 | 37.982 |
| Tubulin alpha-1B chain | TUBA1B | tr\|B3KT06 | 37.519 |
| Emerin | EMD | sp\|P50402 | 36.895 |
| ADP-ribosylation factor-like protein 6-interacting protein 4 | ARL6IP4 | tr\|F5GYV5 | 36.642 |
| Tubulin beta-4B chain | TUBB2C | tr\|Q8N6N5 | 36.102 |
| 60S acidic ribosomal protein P2 | RPLP2 | tr\|A0A024RCA7 | 35.892 |
| Insulin-like growth factor 2 mRNA-binding protein 1 | IGF2BP1 | sp\|Q9NZI8 | 35.802 |
| Neuroblast differentiation-associated protein AHNAK | AHNAK | sp\|Q09666 | 35.24 |
| 60S ribosomal protein L13 | RPL13 | tr\|Q6NZ55 | 34.915 |
| Annexin;Annexin A2 | HEL-S-270 | tr\|V9HW65 | 33.996 |
| High mobility group protein HMG-I/HMG-Y | HMGA1 | sp\|P17096 | 33.769 |
| Nck-associated protein 1 | NCKAP1 | sp\|Q9Y2A7 | 33.656 |
| Calmodulin-like protein 5 | CALML5 | sp\|Q9NZT1 | 33.583 |
| ATPase family AAA domain-containing protein 3A | ATAD3A | sp\|Q9NVI7 | 33.136 |
