# Supplementary material for: CAF-derived exosomal WEE2-AS1 facilitates colorectal cancer progression via promoting degradation of MOB1A to inhibit the Hippo pathway
Source: Cell Death Dis. 2022 Sep 19;13(9):796. doi: 10.1038/s41419-022-05240-7 (PMC9485119; doi:10.1038/s41419-022-05240-7)
Supplement: Supplementary file 10 — Supplementary figure legends [file 41419_2022_5240_MOESM10_ESM.docx]

**Supplementary figure legends**

**Fig. S1** Extraction and identification of fibroblasts and sEVs. **A.** Microscopy observation of primary CAFs and NFs, (scale bar, 100 nm). **B.** sEVs labeling and tracing, (scale bar, 100 nm). **C.** A CCK8 assay detection of cell viability. Data are shown as mean ± SD of three independent experiments. *P < 0.05.

**Fig. S2** **A, B.** CAFs-derived sEVs promote the growth of CRC cells in vitro. Data are shown as mean ± SD of three independent experiments.

**Fig. S3** **A.** Level of LINC00326, miR-3960, miR-4449, miR-6087, miR-4508, and miR-4532 in four paired NF-sEV and CAF-sEV, as determined by qRT-PCR. Data are shown as mean ± SD of three independent experiments. *P < 0.05, **P < 0.01, ns. not signiﬁcant.

**Fig. S4** **A-F.** Level of WEE2-AS1 in CAF, CAF-sEV, HCT 116 and HT-29 (CAF-sEV fed). Data are shown as mean ± SD of three independent experiments.

**Fig. S5** **A-D.** Effect of sEVs WEE2-AS1 on CRC cellular phenotype. Data are shown as mean ± SD of three independent experiments.

**Table S1** Correlation between sEV WEE2-AS1 expression and clinicopathological characteristics of CRC patients.

**Table S2** List of primary antibodies used in the study.

**Table S3** Sequences of primers and shRNAs.

**Table S4** Mass spectrometry assay revealed 71 differential proteins.
